# Nanotechnology-Driven Delivery of Caffeine Using Ultradeformable Liposomes-Coated Hollow Mesoporous Silica Nanoparticles for Enhanced Follicular Delivery and Treatment of Androgenetic Alopecia

**DOI:** 10.3390/ijms252212170

**Published:** 2024-11-13

**Authors:** Nattanida Thepphankulngarm, Suwisit Manmuan, Namon Hirun, Pakorn Kraisit

**Affiliations:** 1 Thammasat University Research Unit in Smart Materials and Innovative Technology for Pharmaceutical Applications (SMIT-Pharm), Faculty of Pharmacy, Thammasat University, Pathumthani 12120, Thailand; nattanida.t@gmail.com (N.T.); namon.hi@tu.ac.th (N.H.); 2 Division of Pharmacology and Biopharmaceutical Sciences, Faculty of Pharmaceutical Sciences, Burapha University, Chonburi 20131, Thailand; suwisit@go.buu.ac.th

**Keywords:** caffeine, ultradeformable liposomes, hollow mesoporous silica nanoparticles (HMSNs), follicular delivery, androgenetic alopecia, confocal laser scanning microscopy (CLSM), hair follicle dermal papilla cells (HFDPCs)

## Abstract

Androgenetic alopecia (AGA) is caused by the impact of dihydrotestosterone (DHT) on hair follicles, leading to progressive hair loss in men and women. In this study, we developed caffeine-loaded hollow mesoporous silica nanoparticles coated with ultradeformable liposomes (ULp-Caf@HMSNs) to enhance caffeine delivery to hair follicles. Caffeine, known to inhibit DHT formation, faces challenges in skin penetration due to its hydrophilic nature. We investigated caffeine encapsulated in liposomes, hollow mesoporous silica nanoparticles (HMSNs), and ultradeformable liposome-coated HMSNs to optimize drug delivery and release. For ultradeformable liposomes (ULs), the amount of polysorbate 20 and polysorbate 80 was varied. TEM images confirmed the mesoporous shell and hollow core structure of HMSNs, with a shell thickness of 25–35 nm and a hollow space of 80–100 nm. SEM and TEM analysis showed particle sizes ranging from 140–160 nm. Thermal stability tests showed that HMSNs coated with ULs exhibited a Td_10_ value of 325 °C and 70% residue ash, indicating good thermal stability. Caffeine release experiments indicated that the highest release occurred in caffeine-loaded HMSNs without a liposome coating. In contrast, systems incorporating ULp-Caf@HMSNs exhibited slower release rates, attributable to the dual encapsulation mechanism. Confocal laser scanning microscopy revealed that ULs-coated particles penetrated deeper into the skin than non-liposome particles. MTT assays confirmed the non-cytotoxicity of all HMSN concentrations to human follicle dermal papilla cells (HFDPCs). ULp-Caf@HMSNs promoted better cell viability than pure caffeine or caffeine-loaded HMSNs, highlighting enhanced biocompatibility without increased toxicity. Additionally, ULp-Caf@HMSNs effectively reduced ROS levels in DHT-damaged HFDPCs, suggesting they are promising alternatives to minoxidil for promoting hair follicle growth and reducing hair loss without increasing oxidative stress. This system shows promise for treating AGA.

## 1. Introduction

Androgenetic alopecia (AGA) is characterized by the impact of the androgen hormone dihydrotestosterone (DHT) on hair follicle regeneration, leading to male and female pattern hair loss (MPHL and FPHL, respectively) [1,2,3]. In specific scalp regions, DHT shortens the anagen phase, thereby inhibiting hair growth [4,5,6]. External factors such as stress and poor nutrition can also contribute to hair loss [7,8]. Currently, FDA-approved treatments include oral finasteride and topical minoxidil (MNX), each with various mechanisms and limitations [9,10,11,12,13]. Potential natural alternatives that promote hair growth and reduce hair loss include azelaic acid, olive oil, garlic gel, and extracts from rice bran and coffee pulp [6,14,15,16].

Caffeine (Caf), a xanthine alkaloid commonly found in plants like coffee, tea, and chocolate, provides numerous benefits for skin health, primarily due to its antioxidant properties. It has also shown potential in the treatment of alopecia by inhibiting the conversion of testosterone to DHT, which is a key contributor to hair follicle shrinkage [17,18]. Nanoparticle-based delivery systems can enhance the effectiveness of Caf treatments for AGA by improving transdermal absorption. Various formulations, including nanostructured lipid carriers (NLCs) [19], liposomes [20], and solid lipid nanoparticles (SLNs) [21], have shown potential for facilitating Caf penetration through the skin barrier. For instance, NLCs enhance Caf’s solubility and stability, increasing its bioavailability and promoting hair growth. Studies have indicated that Caf-loaded NLCs significantly improve skin absorption and stimulate higher hair growth rates than conventional formulations [22]. SLNs, on the other hand, provide controlled release profiles, ensuring sustained Caf delivery to hair follicles. Research suggests that SLNs improve the transdermal delivery of Caf, resulting in more effective therapeutic outcomes in hair loss treatment [21]. Additionally, liposomal formulations can encapsulate Caf, protect it from degradation, and promote its targeted release. For example, a study showed that liposomes containing Caf improved penetration through the stratum corneum and deeper skin layers, which led to enhanced hair growth compared to free Caf [20].

Nanotechnology has greatly improved drug delivery methods, particularly for topical and transdermal treatments. Nanoparticles, ranging from 10 to 1000 nm, enhance skin absorption [23]. Liposomes, discovered by Dr. Alec D. Bangham in the 1960s, are spherical vesicles with a hydrophilic core and phospholipid bilayers, known for their biocompatibility, flexibility, and high encapsulation efficiency, making them excellent drug carriers [24,25,26].

A significant development in this field is the introduction of ultradeformable liposomes (ULs), which exhibit greater elasticity and flexibility than traditional liposomes. These characteristics allow ULs to penetrate the stratum corneum more effectively, improving the distribution of encapsulated drugs like Caf. The deformability of ULs results from their activated edges, facilitating easier passage through skin barriers. Key components such as phospholipids and permeation enhancers, including oleic acid, enhance drug delivery. Studies have demonstrated that ULs surpass conventional liposomes in skin permeation. For instance, Nayak et al. [27] demonstrated ULs containing oleic acid provided better skin penetration than traditional formulations, while Essa et al. [28] showed that ULs improved transdermal drug absorption. These findings underscore the potential of ULs for more efficient drug delivery [29]. Nevertheless, challenges remain, including vesicle fusion, aggregation, and ensuring stability [30]. Liposome stability and homogeneity are essential for maintaining consistent efficacy, particularly when managing hydrophilic substances like Caf and particle size [31,32].

On the other hand, mesoporous silica nanoparticles (MSNs) are highly versatile drug carriers characterized by tunable pore sizes, large surface areas, and excellent biocompatibility. They can be synthesized using alkoxysilanes such as tetraethoxysilane (TEOS) and tetramethoxysilane (TMOS), influencing their physicochemical properties. Research has shown that MSNs can efficiently retain hydrophilic substances, including drugs and biomolecules, due to their large surface area and adjustable pore structures. For example, a study has indicated that MSNs achieve significant loading capacities for hydrophilic drugs while maintaining controlled release profiles [33]. However, challenges such as aggregation and stability during storage remain. To address these issues, hollow mesoporous silica nanoparticles (HMSNs) were developed. HMSNs provide improved drug loading capacity and release kinetics because of their hollow structure, which allows for greater therapeutic agent encapsulation while reducing aggregation risk [34,35,36]. Thus, HMSNs are superior carriers due to their biocompatibility, stability, and chemical inertness [37]. Their adjustable morphology, pore size, and particle size offer excellent control over drug delivery, enhancing stability, controlled release profiles, and encapsulation efficiency [38,39].

HMSNs are distinguished by their ability to enhance the loading and diffusion of bioactive materials due to their low-density structure and high surface-to-volume ratio [40,41,42]. With a robust mesoporous silica core, HMSNs improve the stability of liposomes, ensuring uniform particle size and loading capacity [25]. This structure offers several advantages over traditional MSNs, such as higher drug loading capacity and enhanced stability during transport. The hollow core of HMSNs allows for greater storage of therapeutic agents, making them particularly well-suited for drug delivery applications. Research supports the efficacy of HMSNs in various therapeutic contexts. For example, Xia et al. [43] demonstrated that HMSNs could be functionalized with amine groups to achieve a 28.89% loading capacity for the controlled release of the anticancer drug 5-fluorouracil. Additionally, Guo et al. [34] found that HMSNs exhibit high loading efficiency for oppositely charged hydrophilic drugs, further illustrating their versatility as drug delivery vehicles. Furthermore, Li et al. [44] review highlighted HMSNs’ potential in cancer therapy, noting their ability to improve drug loading, improve targeting efficiency, and minimize side effects on healthy tissues.

Numerous studies have investigated pharmaceuticals encapsulated in liposome-encapsulated MSNs due to their high efficacy as a drug delivery vehicle. Wang et al. developed a dual-coated drug delivery system, doxorubicin hydrochloride-loaded MSNs coated with dual-film of calcium carbonate and lipid bilayer, for anti-tumor therapy [45]. This system shows potential as a targeted and efficient platform, offering delayed drug release, reduced side effects, and improved biocompatibility compared to conventional antitumor drugs. Rathnayake et al. focused on developing a dual-targeted drug delivery nanoassembly for cancer therapy. MSNs were loaded with paclitaxel and coated with a liposomal layer containing DPPC, DOPE, DSPE-PEG (2000) amine, and cholesterol. This formulation enhances drug retention and minimizes leakage. Their study demonstrated an 8-fold improvement in therapeutic efficacy by improving drug delivery and reducing side effects [46]. In addition, Al Mahrooqi et al. also investigated thiolated and PEGylated organosilica nanoparticles for drug delivery, showing the potential to enhance efficacy and reduce side effects [47]. Based on these findings, our research group suggests that liposome-encapsulated HMSNs might have special abilities to treat alopecia by supplying Caf to hair follicle cells. To our knowledge, no prior work has explored this approach. Therefore, the topic of our current investigation is this strategy.

This study introduces a novel application of ultradeformable liposome-coated hollow mesoporous silica nanoparticles to treat alopecia. HMSNs were synthesized using the sol-gel method and template-assisted synthesis, followed by selective etching. Caf encapsulated in liposome, HMSNs (Caf@HMSNs), and ultradeformable liposome-coated HMSNs (ULp-Caf@HMSNs) were investigated. For the liposome-coated HMSNs (Lp-HMSNs) containing oleic acid, the amounts of polysorbate 20 (Tween^®^20) and polysorbate 80 (Tween^®^80) were varied to assess the properties of the resulting ULp-Caf@HMSNs. The primary aim of this study was to evaluate the drug delivery capabilities of various carriers, including nanoparticles, liposomes, and their combinations, while assessing the effects of different polysorbate types (non-ionic surfactants used in ULs) on liposome performance. This approach also aims to optimize follicle penetration and biocompatibility through the liposome coating, enable the controlled release of Caf, and improve molecular stability during transport. By optimizing these parameters, this research seeks to demonstrate the potential of ULp-Caf@HMSNs in enhancing drug efficacy while minimizing side effects. Physical properties such as particle size, pore size, and volume were characterized using a Zetasizer and Brunauer-Emmett-Teller (BET) analysis, while functional groups were identified using Fourier transform infrared spectroscopy (FT-IR). The drug release was tested in phosphate-buffered saline (PBS) at pH 7.4, mimicking hair follicle conditions. Skin penetration ability was evaluated using porcine skin, with visualization of nanoparticle deposition and permeation pathways conducted via confocal laser scanning microscopy (CLSM). This experiment compared the permeation ability of liposomal versus non-liposomal particles. Cell morphology and aggregative behavior were also assessed to evaluate the potential for hair growth, comparing pure Caf with Caf-loaded liposomal and non-liposomal particles. Cytotoxicity was tested through the MTT assay on human follicle dermal papilla cells (HFDPCs). A DCF-DA reactive oxygen species (ROS) assay was used to measure the level of ROS production in HFDPC cells, using 2,7-dichlorofluorescein diacetate (DCF-DA) as a ROS indicator.

## 2. Results and Discussion

### 2.1. Characterization of HMSNs, ULpTW-HMSNs and Derivatives

**Figure 1:**
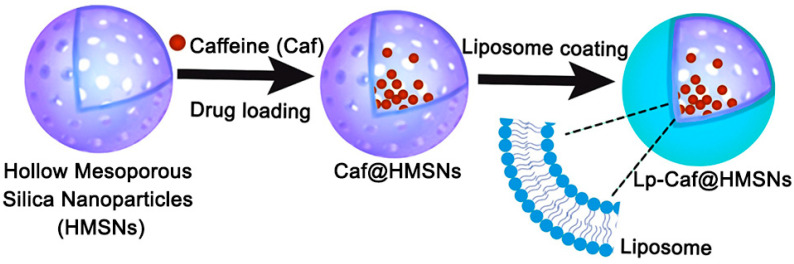
Schematic diagram of the formation of Caf@HMSNs, Lp-Caf@HMSNs, and derivatives.

**Figure 2:**
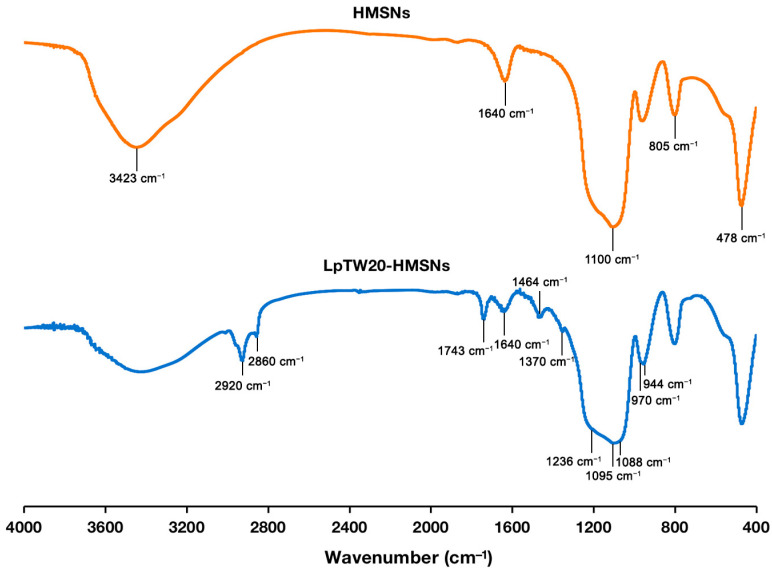
The FT-IR spectra of HMSNs and LpTW20-HMSNs.

Figure 1 illustrates the formation of the Caf@HMSNs, Lp-Caf@HMSNs, and derivatives. Initially, the particles were synthesized and then loaded with Caf. Finally, the outermost layer of the particles was coated with liposomes. Samples without Caf, particularly HMSNs and ULpTW-HMSNs, underwent an initial examination to investigate their functional groups, particle morphology, structural characteristics, and physical properties. FT-IR spectroscopy was used to examine the functional groups of HMSNs and LpTW20-HMSNs, as shown in Figure 2. In the HMSNs spectrum, the hydroxyl O-H stretching and O-H bending bands were observed at approximately 3423 cm^−1^ and 1640 cm^−1^, respectively. The peak at 805 cm^−1^ is attributed to the stretching vibrations of the -Si-O- group, while the strong bands found at 1100 cm^−1^ and 478 cm^−1^ are the stretching and rocking vibrations of the -Si-O-Si- group [48]. Meanwhile, the LpTW20-HMSNs spectra highlighted polysorbate 20’s additional peaks at 2920 cm^−1^ and 2860 cm^−1^, associated with methylene’s symmetric and asymmetric stretching vibrations. Symmetric and asymmetric carbonyl stretching vibrations exhibit peaks at 1370 cm^−1^ and 1640 cm^−1^, respectively. The peak at 1095 cm^−1^ is attributed to the stretching vibrations of -CH_2_-O-CH_2_- [49]. Oleic acid’s characteristic peaks appear at 1464 cm^−1^ and 944 cm^−1^, attributed to the in-plane and out-of-plane stretching vibrations of O–H, respectively. The peaks at 1743 cm^−1^ are attributed to the asymmetric stretching vibration of the carbonyl group [50]. Furthermore, the Phospholipon^®^ 90 G characteristic peaks were identified at 1236 cm^−1^, 1088 cm^−1^, and 970 cm^−1^. These peaks are assigned to the stretching vibrations of P=O, P–O–C, and −N(CH_3_)^3+^, respectively [51]. These FT-IR results demonstrated that the liposome coating on the HMSNs was successful.

**Figure 3:**
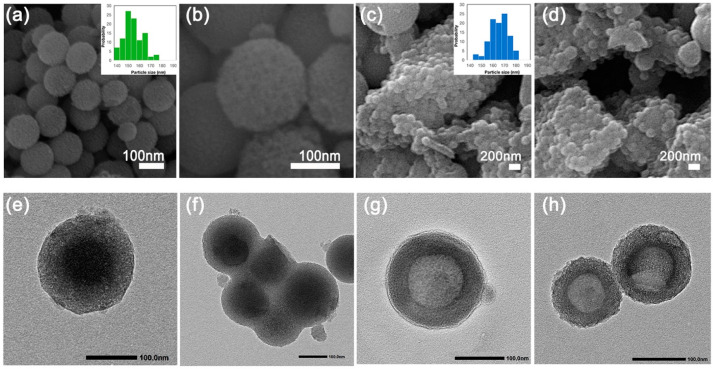
FE-SEM images of (**a**,**b**) HMSNs and (**c**,**d**) LpTW20-HMSNs (Insets: distribution of particle size images). TEM images of (**e**,**f**) SiO_2_@CTAB-SiO_2_ Core/Shell Nanoparticles and (**g**,**h**) HMSNs after etching.

Scanning electron microscopy (SEM) images of HMSNs and LpTW20-HMSNs are shown in Figure 3a,b and Figure 3c,d, respectively. The images demonstrate the spherical shape and monodispersed nature of the prepared particles. The diameter of the HMSNs was found to be between 140 and 160 nm, indicating the production of particles at the nanoscale. However, the LpTW20-HMSN particles appeared to be larger, by approximately 20–30 nm, due to the additional outer layer of liposomes. Figure 3e–h shows transmission electron microscopy (TEM) images of the HMSNs. TEM images of HMSNs without the etching process are shown in Figure 3e,f. These images reveal the hard templates of SiO_2_ nanoparticles, indicated by the dark core at the center of the particles. Following the etching procedure, the representative TEM images of HMSNs displayed in Figure 3g,h demonstrate the mesoporous shell structures and space at the core, suggesting the removal of the rigid template. After the liposome coating (LpTW20-HMSN), an additional thin layer was observed on the outer surface, confirming that the particles were encapsulated within the liposome (Figure S1). Measurements showed that the MSN shell thickness was approximately 25–35 nm, while the hollow space width was about 80–100 nm. Both the hollow cavity and the MSN shell thickness are readily tunable.

Table 1 illustrates the zeta potential, PDI, and average particle size of LpTW20-HMSNs, LpTW80-HMSNs, LpTW2080-HMSNs, Lp-HMSNs, HMSNs, and LpTW20. The produced samples ranged in size from 78 to 183 nm. LpTW20-HMSNs, LpTW80-HMSNs, LpTW2080-HMSNs, and Lp-HMSNs showed average particle sizes between 170 and 183 nm, which are considerably larger than those of the HMSNs (which have no liposome coating). A minor decrease in particle size was observed in formulations of LpTW2080-HMSNs and LpTW80-HMSNs with added surfactant. This reduction aligns with previous studies indicating that surfactants reduce the interfacial tension between the lipid bilayer and the surrounding aqueous phase. This reduction promotes improved emulsification and uniform liposome dispersion, leading to smaller sizes [52,53]. However, the LpTW20-HMSNs formulation containing polysorbate 20 showed a larger particle size than the LpTW80-HMSNs and LpTW2080-HMSNs formulations containing polysorbate 80. This difference is likely due to the greater hydrophobicity of polysorbate 80 (with an HLB value of approximately 15.0) compared to polysorbate 20 (HLB around 16.7) [54], resulting in smaller particle sizes. Surfactants with longer carbon chains or multiple hydrophobic groups are more effective at embedding within the lipid bilayer. This embedding increases the curvature of the bilayer, promoting the formation of smaller vesicles. Highly hydrophobic surfactants are especially likely to create smaller vesicles due to their strong anchoring within the lipid structure [52,55]. Moreover, the LpTW20, which consisted only of liposome nanoparticles without HMSNs, was the smallest. This could indicate that HMSNs increase the samples’ particle sizes and pore volume. The permeability coefficient associated with liposome size suggests that liposome size has a significant influence. In addition, the deeper epidermal layers showed the largest reservoir and the highest drug concentration in the reservoir at an intermediate particle size of 300 nm. Compared to larger liposomes, smaller ones exhibited the most accumulation in the stratum corneum (SC) and enhanced penetration into the deeper skin layers [56,57,58]. Consequently, this study’s HMSNs, Lp-HMSNs, and derivative particles were within the optimal size range. The samples may be able to incorporate Caf into the target cells.

The average particle size, as determined by dynamic light scattering (DLS), TEM, and SEM techniques, correlates well across each technique and falls within the optimal range for permeability in the deeper epidermal layers. This suggests a high capacity for drug reservoirs and a maximum concentration of medication. Consequently, these particles constitute an effective drug delivery system for delivering Caf to the target cells.

The generated LpTW20-HMSNs, LpTW80-HMSNs, LpTW2080-HMSNs, Lp-HMSNs, HMSNs, and LpTW20 had a size distribution within an acceptable range, as indicated by the PDIs being less than 0.7 [59], and the zeta potential was between −26.2 and −33.7 mV. According to Méndez et al. [60], the zeta potential of HMSNs revealed a negative charge because their pKa is 3.5–4.6, and they ionize to form negatively charged molecules at neutral pH 7 (degassed DI water). A stronger negative charge was observed in Lp-HMSNs compared to HMSNs. The reason is that the zwitterionic molecule phosphatidylcholine, which contains a positively charged choline group and a negatively charged phosphate group, gives the Lp-HMSNs a stronger negative charge. Its isoelectric point is approximately 6 [61]. Furthermore, because of the oleic acid, LpTW20-HMSNs, LpTW80-HMSNs, LpTW2080-HMSNs, and LpTW20 displayed the highest negative charge. Due to their carboxylic groups, the fatty acids were ionized to form negatively charged molecules. As a result, this would support raising the negative charge. The negative charge of the samples is crucial for their electrostatic stability; this repulsive force keeps the particles from aggregating and preserves their colloidal stability. Further, the negative charge promotes advantageous interactions with the skin’s surface, especially the outermost layer’s stratum corneum. This charge probably encourages skin surface adherence, which is important for combining the lipid components of the stratum corneum, to the skin barrier. This interaction may increase the delivery of both hydrophilic and lipophilic drugs by allowing the chemicals to pass through the epidermal barrier [62] more effectively. The results might indicate that a special liposome with a larger negative charge could facilitate easier particle penetration.

**Figure 4:**
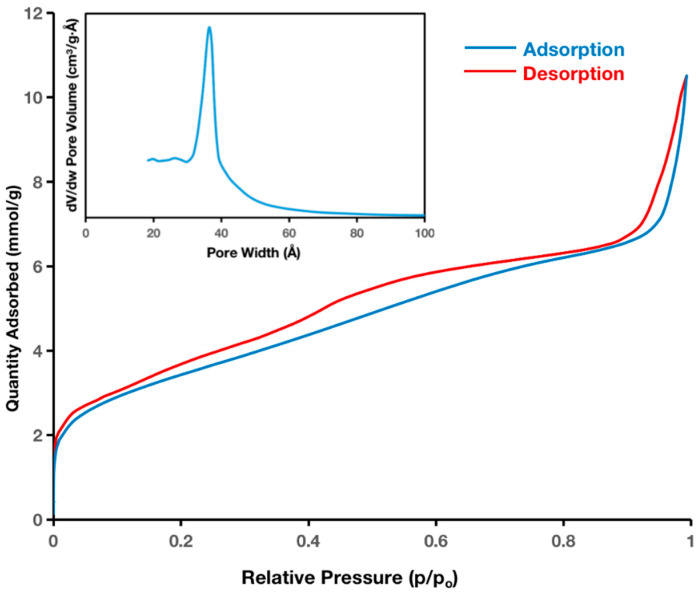
The adsorption–desorption isotherms of HMSNs (lower curve in blue is adsorption, the upper curve in red is desorption; insets show pore size distributions).

The N_2_ sorption isotherms of the HMSNs were used to calculate their pore diameters, pore volumes, and specific surface areas. According to IUPAC categorization, the adsorption–desorption isotherms in Figure 4 are type IV isotherms, which are typical of mesoporous materials [63]. According to their pore size distributions (Figure 4 inset), these HMSNs had average pore sizes of 3.6 nm, within the range of mesoporous materials. Compared to conventional MSNs, HMSNs exhibit a higher pore volume of 1.11 mL g^−1^, suggesting the presence of hollow spaces that contribute to the increased pore capacity. Additionally, the specific surface area measured by BET analysis was 1044 m^2^ g^−1^. With particle sizes below 200 nm, our HMSNs showed a larger pore volume than typical MSNs [64]. Compared to other studies, the pore volume of HMSNs surpassed that of conventional MSNs, which have reported values of 0.69 mL g^−1^ [65] and 0.47–0.60 mL g^−1^ [66]. These findings further support the notion that the hollow structure of HMSNs enhances their pore volume, making them suitable for high-capacity drug delivery systems.

**Figure 5:**
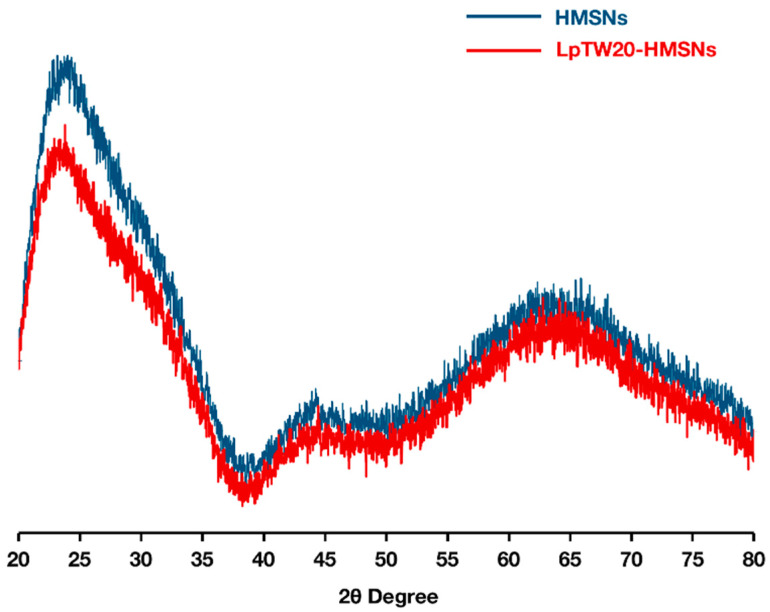
Powder XRD patterns obtained from the HCl/ethanol extraction method for as-synthesized HMSNs and LpTW20-HMSNs (lower curve in red is LpTW20-HMSNs and upper curve in blue is HMSNs).

An X-ray diffraction (XRD) analysis was conducted on the powder samples, and the resulting diffraction patterns are presented in Figure 5. HMSNs are generally considered amorphous due to their synthesis methods, such as sol-gel processes, which typically produce non-crystalline structures. This amorphous nature contributes to HMSNs’ high surface area and porosity, making them ideal for drug delivery and catalysis applications. The diffraction pattern of the hollow core–shell silica nanoparticles exhibits a characteristic silica peak at 2θ = 23°, as reported by Deepika et al. [67], with broad peaks confirming the amorphous structure. Additionally, our results indicate that the pore structure of the original material was preserved during the template removal process using HCl–ethanol extraction [68]. Furthermore, the LpTW20-HMSNs samples displayed the same characteristic peaks as HMSNs after liposome coating, suggesting that the ULs did not affect the structural stability of the HMSNs.

**Figure 6:**
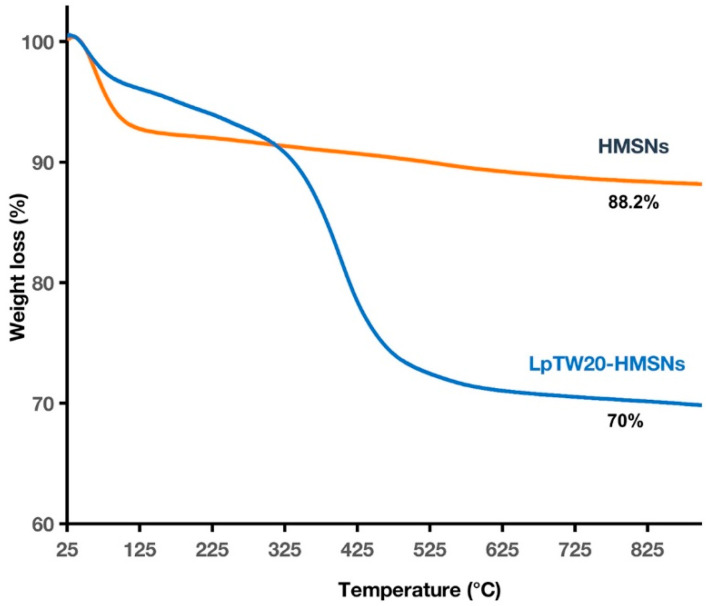
TGA analysis of HMSNs and LpTW20-HMSNs.

The thermal stability of HMSNs was evaluated based on their 10% weight-loss temperatures (Td_10_) values and char yields at 900 °C. The Td_10_ value was approximately 375 °C, with a residual ash of 88.2%, indicating good thermal stability. The thermogravimetric analysis (TGA) pattern of HMSNs (Figure 6) shows an initial weight loss of about 8–9% from room temperature to around 125 °C, attributed to the thermodesorption of physically adsorbed water and impurities [69]. Beyond this, up to 900 °C, only minor weight loss was observed. A minor weight loss of approximately 4–5% within this range was due to the decomposition of residual CTAB surfactant and oxygen functional groups on the particles [70]. The thermal study of LpTW20-HMSNs showed a Td_10_ value of approximately 325 °C, with residual ash of 70%. Although LpTW20-HMSNs exhibited lower thermal stability than HMSNs, they still demonstrated good thermal stability. An initial weight loss of about 3–4% from 25 °C to below 100 °C was attributed to the removal of residual water or volatile components in the liposomes during heating. Up to 350 °C, a total loss of 6–7% suggested relative stability within this temperature range, indicating that the liposomes maintained their structural integrity under thermal stress. Significant structural changes occurred between 350–550 °C due to increased thermal stress, leading to further degradation of the lipid bilayers [71]. The liposomes completely decomposed at temperatures exceeding 550 °C, leaving only HMSNs. From these findings, both HMSNs and LpTW20-HMSNs were stable within the temperature ranges used in the experiments.

### 2.2. Caf Loading Efficiency and Releasing Profiles of Caf@HMSNs, ULp-Caf@HMSNs, and Derivatives

Table 2 shows the effect of solvents on the % entrapment efficiency (%EE) and % loading capacity (%LC) of Caf-loaded HMSNs, yielding 36.01% EE and 26.47% LC in water and 32.10% EE and 24.32% LC in ethanol, respectively. This suggests that water, a highly polar solvent, enhances Caf’s solubility due to its polar nature and ability to form hydrogen bonds. Water’s high dielectric constant (~80) stabilizes ions and polar molecules [72]. Conversely, ethanol’s hydrophobic ethyl group interacts unfavorably with the silanol groups of HMSNs and Caf’s polar regions, reducing solubility. Additionally, water minimizes hydrophobic interactions, improving stability within the HMSN cavity. Water is also preferred for its non-toxicity, ease of handling, and compatibility in pharmaceutical and food industries, ensuring safe drug delivery. The %EE of Caf@HMSNs was compared with Caf@LpTW20, revealing that Caf@LpTW20 had a 5–6% lower %EE. This indicates that HMSNs, with their larger pore volume and greater stability in various biological environments, allow for a higher %LC. Consequently, Caf loading in HMSNs using water as the solvent was selected for further study.

Furthermore, the ability to load Caf into nanoparticles was compared with previous work. The capacity to contain Caf is shown in Table S1. The experimental results indicate that most previous nanoparticles had a %LC of less than 25%. In our experiment, the HMSNs achieved an LC value as high as 26%. This suggests that HMSNs have a larger cavity volume and can carry more Caf, making them suitable for delivering Caf into hair follicles.

**Figure 7:**
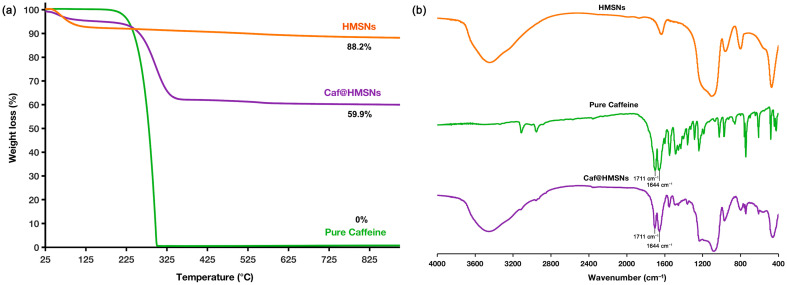
The images of (**a**) TGA analysis and (**b**) FT-IR spectra of pure Caf, HMSNs, and Caf@HMSNs.

The quantity of Caf loaded was further verified using TGA and FT-IR spectroscopy. Figure 7a presents the thermogravimetric curves for pure Caf and Caf@HMSNs. Pure Caf decomposed within the temperature range of 200–305 °C, while the Caf within the HMSNs decomposed at a slightly higher range of 225–355 °C. This increase in decomposition temperature suggests that the outer layer of the HMSNs protected the encapsulated Caf. The residue ash was 59.9%, indicating that approximately 40.1% of the total weight loss was attributed to decomposition, of which about 28.3% was due to Caf. Figure 7b confirms Caf loading through FT-IR spectra of HMSNs, pure Caf, and Caf@HMSNs. The strong C=O stretching bands observed at 1711 and 1664 cm^−1^ in the FT-IR spectrum of pure Caf were also present in Caf@HMSNs, though with reduced intensity. This reduction in intensity is likely due to Caf’s confinement within the HMSN pores [73]. TGA and FT-IR spectroscopy confirmed that Caf was effectively loaded into the porous HMSNs.

From Equations (4)–(7), the release kinetic data for Caf release are shown in Table 3. The chosen mathematical models to characterize the release pattern were zero-order, first-order, Higuchi, and Korsmeyer–Peppas models. The model selection was based on the maximum correlation coefficient (R^2^) to best analyze the Caf release profiles. The Higuchi model exhibited the highest R^2^ value, suggesting it was the most suitable model for representing the Caf release profiles and indicating a diffusion-controlled release mechanism [74,75,76,77]. The Higuchi model, which describes drug release as a diffusion process based on Fick’s law, is well-suited for delivering active ingredients dispersed in a solid matrix, such as in alopecia treatments. It enables sustained and controlled release to hair follicles, maintaining effective concentrations over time, which is key for treating chronic conditions like alopecia. This model’s diffusion-controlled release minimizes systemic absorption and reduces potential side effects. Additionally, the prolonged release profile supports lower application frequency, enhancing patient compliance and therapeutic outcomes by helping to maintain drug levels within the therapeutic range. For Caf@HMSNs, ULp-Caf@HMSNs, and derivatives, the Higuchi model effectively characterizes diffusion-controlled release, supporting targeted delivery to hair follicles [78,79].

**Figure 8:**
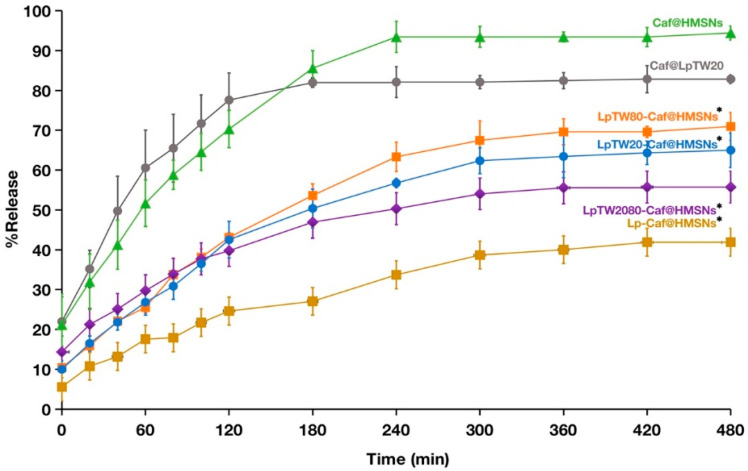
Release behavior of Caf in PBS (pH = 7.4) at 37.5 °C of LpTW20-Caf@HMSNs, LpTW80-Caf@HMSNs, LpTW2080-Caf@HMSNs, Lp-Caf@HMSNs, Caf@HMSNs, and Caf@LpTW20. Data are expressed as mean and SD (*n* = 3). * *p* < 0.05 when compared to Caf@LpTW20 and Caf@HMSNs.

Since the deep-within hair follicle cells have a pH of 7.4, a PBS pH 7.4 solution was selected for this experiment [80]. In Figure 8, the Caf release profiles at eight hours of analysis are displayed for the LpTW20-Caf@HMSNs, LpTW80-Caf@HMSNs, LpTW2080-Caf@HMSNs, Lp-Caf@HMSNs, Caf@HMSNs, and Caf@LpTW20, which were 65%, 71%, 55%, 39%, 94%, and 83%, respectively. The data showed that Caf@HMSNs had the highest drug release percentage, surpassing Caf@LpTW20. This is due to the larger pore volume of HMSNs, allowing a greater %LC. In contrast, liposomes have limited encapsulation efficiency due to their phospholipid bilayer structure. Moreover, HMSNs are more stable in various biological environments, enhancing their drug release performance. However, it was observed that samples LpTW20-Caf@HMSNs, LpTW80-Caf@HMSNs, LpTW2080-Caf@HMSNs, and Lp-Caf@HMSNs exhibited a lower percentage of Caf release compared to the two samples above. The reduction in release may be attributed to the liposome coating, which partially blocked the pores of the HMSNs. This coating forms a barrier around the particles, hindering the release of the Caf within the cavity and leading to a lower overall release percentage. Nearly all samples showed the highest release percentage at the seventh hour, and an initial burst of Caf release was observed in every system. It was observed that Caf@LpTW20 released Caf more quickly than other forms. The system contained only liposomes, which degraded faster than HMSNs [81]. This can sometimes lead to premature drug release or leakage. Drug release from HMSNs involves a combination of diffusion through pores and silica matrix degradation, while liposomes primarily release drugs through fusion with cell membranes or lipid bilayer destabilization. Consequently, Caf@LpTW20 achieved a complete Caf release within three hours. While LpTW20-Caf@HMSNs, LpTW80-Caf@HMSNs, LpTW2080-Caf@HMSNs, and Lp-Caf@HMSNs released the drug more slowly than Caf@HMSNs and Caf@LpTW20, this is due to the dual presence of HMSNs and liposomes in these systems. HMSNs aid in controlled drug release, while the liposomes covering the exterior helped seal some of the pores, preventing Caf from escaping the HMSN particles too quickly. This combination effectively slows down the overall drug release process. Gradual Caf release ensures sustained delivery to cells, prolonging its presence in the body and reducing the need for frequent administration. This controlled release minimizes overdose risk, enhancing patient safety and treatment effectiveness.

Moving on to the four types of HMSNs coated with liposomes, it was observed that Lp-Caf@HMSNs had the lowest percentage of drug release. This is because Lp-Caf@HMSNs, conventional liposomes without polysorbate and oleic acid in the bilayer, do not permit hydrophilic molecules like Caf to permeate unless the liposome structure breaks down [82]. In contrast, ULs, which contain polysorbate and oleic acid in their phospholipid membranes, facilitate interactions that enable the movement of hydrophilic molecules through the bilayer via transbilayer diffusion or flip-flop [83]. Among the ULs groups, LpTW2080-Caf@HMSNs exhibited the lowest drug release. This suggests that micelles formed with a mixture of surfactants are more homogeneous, leading to more predictable and consistent drug delivery. Additionally, the different hydrophilic–lipophilic balance (HLB) values of polysorbate 20 (16.7) and polysorbate 80 (15) can cause complex interactions when combined, potentially reducing the efficiency of micelle formation [84]. It was evident from the drug release experiment that the most suitable delivery system for delivering Caf to the hair follicles was HMSNs covered with ULs.

### 2.3. In Vitro Permeation Studies of Caf@HMSNs, ULp-Caf@HMSNs and Derivatives

**Figure 9:**
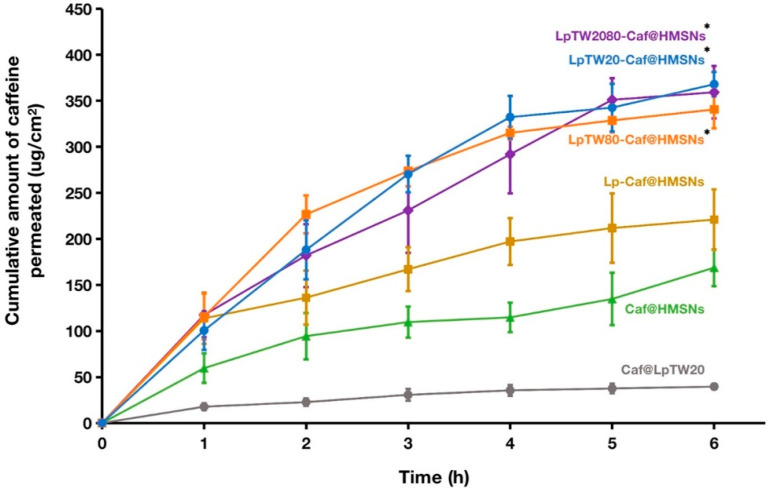
Cumulative amount per area of Caf from LpTW20-Caf@HMSNs, LpTW80-Caf@HMSNs, LpTW2080-Caf@HMSNs, Lp-Caf@HMSNs, Caf@HMSNs, and Caf@LpTW20 though the porcine skin. Data are expressed as mean and SD (*n* = 3). * *p* < 0.01 when compared to Caf@LpTW20.

Figure 9 and Table 4 illustrate in vitro permeation profiles of LpTW20-Caf@HMSNs, LpTW80-Caf@HMSNs, LpTW2080-Caf@HMSNs, Lp-Caf@HMSNs, Caf@HMSNs, and Caf@LpTW20. The cumulative amount per area was used to assess the permeation of Caf through porcine skin from the formulations. Additionally, steady-state flux (J_ss_) and apparent permeability coefficient (P_app_) were used to determine Caf permeation. The J_ss_ is calculated by taking the slope of the linear portion of the permeation curve and dividing it by the surface area of the porcine skin available for permeation [85,86]. The P_app_ is obtained by dividing the steady-state flux by the initial concentration of the drug in the donor solution [85,86]. The findings revealed a consistent trend across the three variables—cumulative amount per area, J_ss_, and P_app_—showing that formulations with ULs permeated porcine skin more effectively than those without. Among these, the LpTW20-Caf@HMSNs had the highest values for all three parameters. This formulation contained polysorbate 20, which is more hydrophilic and has a smaller molecular structure than polysorbate 80. Consequently, formulations with polysorbate 20 enhance permeation and disrupt lipid barriers more effectively than those containing polysorbate 80 [54,87].

Caf@LpTW20 exhibited the lowest Caf penetration through the porcine skin. Based on the results of the Caf@LpTW20 rapid-release experiment, it can be inferred that this formulation releases Caf quickly, leaving a significant amount of Caf on the surface of the porcine skin without effectively penetrating the skin layer. Due to its hydrophilic nature, Caf struggles to permeate the skin, as the lipophilic SC serves as a barrier, resulting in lower penetration than other formulations [88,89]. Although Caf@HMSNs displayed the highest percentage of Caf release, it showed the second lowest Caf penetration, 168.79 μg cm^−2^. This suggests that HMSNs without liposomes exhibit low biocompatibility with cells, resulting in reduced skin penetration.

In contrast, Lp-Caf@HMSNs had a Caf penetration concentration of 221.02 μg cm^−2^, significantly higher than Caf@HMSNs. The presence of liposomes in Lp-Caf@HMSNs increased cell biocompatibility, facilitating particle diffusion through tissues and Caf release. Incorporating Caf into the liposome–HMSNs system effectively masks its hydrophilic properties, allowing for better penetration through the SC. Furthermore, liposomes integrate with cellular membranes, enhancing drug permeation across the skin layers. This makes them efficient carriers for transdermal delivery, increasing drug concentrations within the epidermis and dermis [90,91]. LpTW20-Caf@HMSNs, LpTW80-Caf@HMSNs, and LpTW2080-Caf@HMSNs showed a rapid increase in Caf concentration during the first 4–5 h, leveling off in the last hour. The Caf penetration concentrations for these three samples ranged from 340 to 370 μg cm^−2^, the highest. This suggests that including polysorbate 20 and/or polysorbate 80 in these formulations converts simple liposomes into ULs, enhancing elasticity and flexibility. Consequently, more particles could easily be transported through the SC, improving skin penetration. Thus, combining HMSNs and ULs proved optimal for drug delivery to the target cells. Given that LpTW20-Caf@HMSNs exhibited the greatest total Caf penetration, it was selected for the in vitro experiment.

After 24 h, the porcine skin samples were extracted with water were extracted, and the cumulative amount of drug in the porcine skin is presented in Table 4. The remaining amount of Caf in the porcine skin ranged from 25 to 70 μg, indicating that a small portion remained on the porcine skin. This suggests that HMSNs, particularly those with liposome integration, demonstrate high potential as vehicles for transporting Caf through porcine skin.

**Figure 10:**
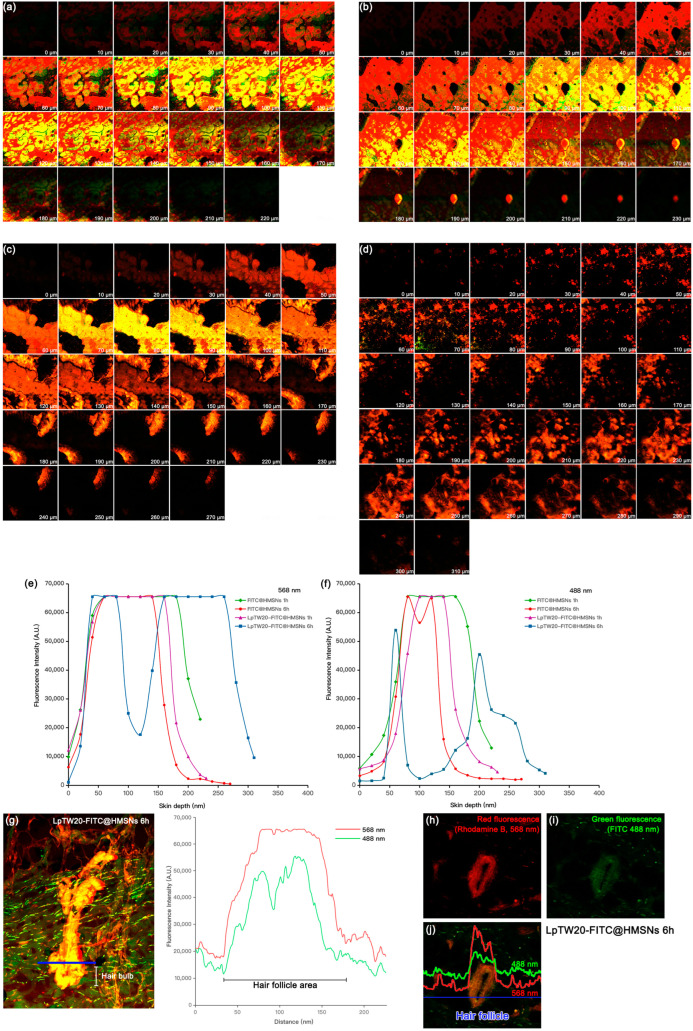
The CLSM images illustrate the penetration of porcine skin after treatment with (**a**) FITC@HMSNs for one hour, (**b**) LpTW20-FITC@HMSNs for one hour, (**c**) FITC@HMSNs for six hours, and (**d**) LpTW20-FITC@HMSNs for six hours. The fluorescence intensity profiles at varying skin depths for (**e**) Rhodamine B (568 nm) and (**f**) FITC (488 nm). The cross-sectional CLSM images of (**g**) hair follicles treated with LpTW20-FITC@HMSNs for six hours, along with the corresponding fluorescence intensity profiles within the hair follicles and the fluorescence intensity profiles across the skin layers for LpTW20-FITC@HMSNs at six hours show (**h**) red fluorescence from Rhodamine B labeling the particles, (**i**) green fluorescence from FITC, and (**j**) a merged image combining (**h**) and (**i**) (10× objective lens).

The visualization of porcine skin deposition and the permeation pathways of nanoparticles was conducted using CLSM. The permeation depths of the nanoparticles are illustrated in Figure 10. FITC@HMSNs and LpTW20-FITC@HMSNs, coated with Rhodamine B, were selected for this experiment to compare the permeation ability of liposomal versus non-liposomal particles. Fluorescein isothiocyanate (FITC) and Rhodamine B were used at concentrations equivalent to Caf (0.1 g in 50 mL solvent). Before starting the test, porcine skin without FITC and Rhodamine B was tested to assess its autofluorescence response. No autofluorescence was detected in the excitation and emission wavelength ranges. The skin layers relevant for drug delivery via skin penetration can be divided into three primary layers: the SC, which is the outermost layer and typically ranges from 10 to 30 μm in thickness; the epidermis, situated below the SC, which can be 50 to 100 μm thick, depending on the body region; the dermis, the thickest layer of the skin, which houses the hair follicles that extend from the epidermis into the dermis, often reaching several millimeters deep into this layer [92]. We hypothesize that these particles can reach the hair bulb, located at the base of the follicle, where hair growth primarily occurs.

After one hour of treatment, FITC@HMSNs and LpTW20-FITC@HMSNs were observed to penetrate to depths of 220 and 230 µm, respectively. Initially, both FITC@HMSNs and LpTW20-FITC@HMSNs were predominantly distributed within the SC and epidermis, as indicated by the strong fluorescence observed at depths of 50–160 µm in Figure 10a,b. Furthermore, the fluorescence peaks for FITC (488 nm) and Rhodamine B (568 nm) were highest during the first hour for both types of particles (Figure 10e,f). However, after six hours, both particles penetrated the skin deeper with maximum depths of 270 µm for FITC@HMSNs and 310 µm for LpTW20-FITC@HMSNs. Figure 10c shows that most FITC@HMSNs remained trapped at depths of 50–160 µm after six hours, similar to their distribution after one hour, suggesting that these particles are limited in their ability to reach deeper layers, including the hair bulb. In contrast, Figure 10d shows that LpTW20-FITC@HMSNs penetrated deeper, with most particles distributed at depths greater than 180 µm. This observation supports the data from the in vitro permeation study, which demonstrated that the cumulative permeation of LpTW20-FITC@HMSNs into the deeper skin layers after six hours was greater than that of FITC@HMSNs. The results also align with previous studies on the role of the infundibulum, which is the uppermost part of the hair follicle and serves as a trans follicular pathway, allowing macromolecular proteins to bypass the SC and facilitate rapid delivery through the hair follicle [93,94]. Liposomes, known for their high biocompatibility, may enhance compatibility with the infundibulum, allowing easier trans follicular delivery.

Additionally, Figure 10e,f confirms the intensity of FITC and Rhodamine B. The intensity for FITC@HMSNs remained similar at 1 and 6 h, while the intensity for LpTW20-FITC@HMSNs increased significantly at deeper levels. Figure 10g shows a cross-section of hair follicles treated with LpTW20-FITC@HMSNs at six hours. Most particles were observed throughout the hair follicle, with strong fluorescence from both wavelengths, especially in the hair bulb. This suggests that LpTW20-FITC@HMSNs are an effective drug delivery system for alopecia, capable of penetrating the skin and delivering the drug to the target cells. Moreover, the release of the encapsulated compound from HMSNs into the hair follicle was further confirmed by Figure 10h–j, demonstrating a higher fluorescence intensity at 488 nm and 568 nm within the hair follicle than the surrounding skin. This observation suggests that loading FITC into the particles enhanced targeted delivery to the desired cells while minimizing release in non-target areas. As a result, this targeted approach reduces potential side effects and enhances treatment efficacy by concentrating the therapeutic agent at the intended site.

### 2.4. Study of Cell Viability, Cell Morphology, Aggregation Behavior, and DCF-DA Reactive Oxygen Species (ROS) Assay

**Figure 11:**
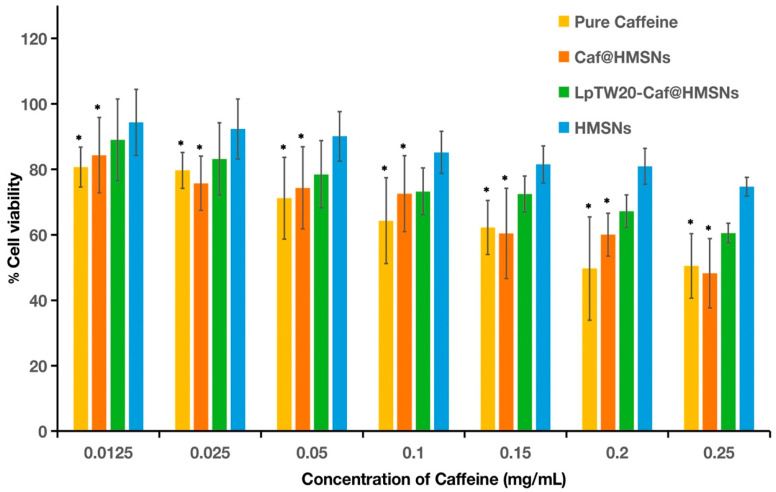
The effect of various Caf concentrations and formulations on HFDPCs viability. Data are expressed as mean and SD (*n* = 3). * *p* < 0.01 when compared to HMSNs.

HFDPCs were treated with Caf for 24 h to assess potential cytotoxicity. An MTT assay was used to evaluate the effects of Caf, HMSNs, Caf@HMSNs, and LpTW20-Caf@HMSNs on cell viability. According to the ISO 10993-5 standard, cell viability must exceed 70% compared to the control to classify a material as non-cytotoxic [95,96]. As depicted in Figure 11, all concentrations of HMSNs showed no cytotoxic effects on HFDPCs, confirming that HMSNs are a promising vehicle for drug delivery. Their large pore volume, high stability, and non-cytotoxic nature make them suitable for transporting Caf to cells. For pure Caf, the MTT assay revealed that certain concentrations (0.0125, 0.025, and 0.05 mg mL^−1^) did not exhibit cytotoxicity in HFDPCs. The findings of this experiment are consistent with those reported by Muangsanguan et al., who used Arabica Coffee Pulp Extracts and observed that the maximum concentration of 0.125 mg mL^−1^ did not exhibit toxicity to the cells [6]. However, at high concentrations, cell viability dropped below 70%, indicating that elevated levels of Caf could induce apoptosis in cells [97]. In contrast, Caf@HMSNs demonstrated higher cell viability at equivalent Caf concentrations than pure Caf. This suggests that HMSNs can modulate Caf release, gradually dispensing it to minimize toxicity. Moreover, LpTW20-Caf@HMSNs exhibited even greater cell viability than pure Caf and Caf@HMSNs, indicating that the liposome component enhances biocompatibility and does not increase cellular toxicity.

**Figure 12:**
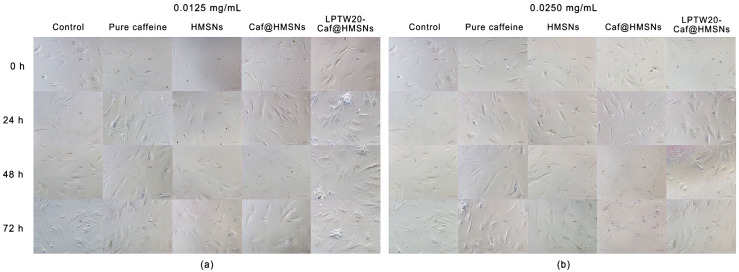
The images of cell morphology and aggregation behavior of HFDPCs treated with Caf at concentrations of (**a**) 0.0125 mg mL^−1^ and (**b**) 0.0250 mg mL^−1^ for various time intervals (0–72 h).

To evaluate the effects of Caf-loaded formulations on HFDPCs, cells were treated with Caf at concentrations of 0.0125 mg mL^−1^ and 0.0250 mg mL^−1^ for 0–72 h, with subsequent examination of their morphology and aggregative behavior (Figure 12). There were no notable changes in cell aggregation behavior in the control group (untreated) and HMSNs-treated cells. HMSNs exhibited no cytotoxicity at these concentrations (cell viability remained above 90%), consistent with MTT assay results. Both groups demonstrated similar cell growth across both concentrations. Cell viability remained around 80% for pure Caf at both concentrations, indicating no cytotoxicity in HFDPCs. Additionally, Caf promoted cell proliferation, as evidenced by increased cell aggregation and larger aggregate sizes in the treated cells compared to the control group. At 0.0125 mg mL^−1^, Caf@HMSNs were non-cytotoxic and showed similar results to pure Caf, though with slightly reduced cell aggregation. However, at 0.025 mg mL^−1^, Caf@HMSNs became toxic (cell viability dropped below 70%), leading to cell death and a significant reduction in aggregation size and number. Interestingly, LpTW20-Caf@HMSNs at 0.0125 mg mL^−1^ exhibited the highest level of cell aggregation, both in size and number. This enhanced performance is attributed to the liposome coating, which improved biocompatibility without increasing toxicity, thereby promoting greater cell growth than pure Caf or Caf@HMSNs alone. This finding underscores the role of liposomes in enhancing drug delivery systems and improving cell viability in therapeutic applications [98]. Thus, the capacity of LpTW20-Caf@HMSNs to promote HFDPC cell aggregation is linked to its potential to stimulate the regeneration of new hair follicles and initiate hair growth.

**Figure 13:**
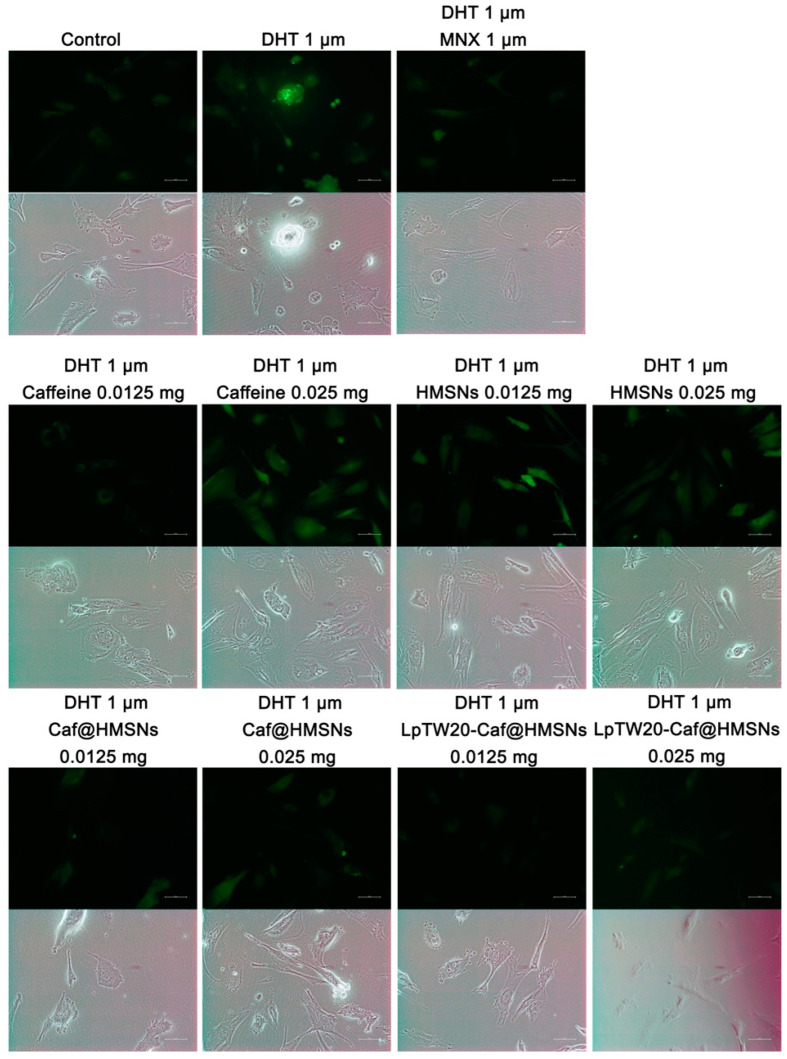
The effects of MNX, 0.0125, and 0.025 mg mL^−1^ concentrations of pure Caf, HMSNs, Caf@HMSNs, and LpTW20-Caf@HMSNs on ROS levels in DHT-damaged HFDPCs were assessed. Fluorescence microscopy was used to capture DCF-DA images, where the intensity of green fluorescence correlates with ROS concentration.

Reactive oxygen species (ROS) are chemical compounds formed within cells in response to internal or external stimuli, and their excessive buildup can lead to cellular damage and various diseases [95,99]. Previous studies have shown that dihydrotestosterone (DHT) induces the production of intracellular ROS in HFDPCs [95,100]. To assess the levels of ROS in HFDPCs, the 2,7-dichlorofluorescein diacetate (DCF-DA) assay was employed (Figure 13). As expected, ROS levels were elevated in the group treated with DHT alone and in the groups treated with DHT in combination with HMSNs at concentrations of 0.0125 mg mL^−1^ and 0.025 mg mL^−1^. This suggests that HMSNs, when used as drug carriers, do not possess inherent antioxidant properties. In contrast, ROS levels were reduced in the DHT-treated group supplemented with MNX. Additionally, pure Caf at a concentration of 0.0125 mg mL^−1^ demonstrated potential in reducing ROS production, exhibiting effects comparable to those observed in the MNX-treated group. However, at a higher concentration of 0.025 mg mL^−1^, Caf increased ROS production in HFDPCs exposed to DHT, indicating that excessive Caf can induce metabolic stress due to high concentrations [101,102]. At concentrations of 0.0125 and 0.025 mg mL^−1^, Caf@HMSNs and LpTW20-Caf@HMSNs markedly reduced ROS levels in DHT-damaged HFDPCs, comparable to the effects of MNX treatment. This effect was particularly notable for both concentrations of LpTW20-Caf@HMSNs. These samples contained the same Caf concentration as the pure Caf group but did not produce elevated ROS. This suggests that the encapsulation of Caf within HMSNs enabled controlled release, preventing excessive ROS generation. Thus, LpTW20-Caf@HMSNs’s ability to inhibit ROS production is advantageous for promoting hair follicle cell growth and reducing hair loss, offering a potential alternative to MNX without the associated side effects [103].

In summary, LpTW20-Caf@HMSNs demonstrate non-toxicity, effective permeation, and controlled drug release, suggesting promising therapeutic use. The clinical application of LpTW20-Caf@HMSNs for alopecia treatment faces challenges, particularly with cost, scalability, and regulatory requirements [104]. Although synthesis involves expensive materials and advanced techniques, LpTW20-Caf@HMSNs may offer a more affordable, targeted, and pain-free alternative to existing hair loss treatments in the long term. Scaling production from lab to industry introduces complexities in ensuring nanoparticle consistency. However, LpTW20-Caf@HMSNs have shown stability and scalability, supporting their industrial potential. Regulatory approval is also essential, requiring comprehensive safety, toxicity, and biodistribution data. Future studies will focus on advancing toward human clinical trials and evaluating their therapeutic potential.

## 3. Materials and Methods

### 3.1. Chemicals and Materials

Sodium carbonate (Na_2_CO_3_), Tetraethyl Orthosilicate (TEOS), Cetyltrimethylammonium bromide (CTAB), Cholesterol (C_27_H_46_O, stabilized with alpha-tocopherol), cis-9-Octadecenoic acid (Oleic acid), Fluorescein 5-Isothiocyanate (FITC Isomer I), Rhodamine B (C_28_H_31_ClN_2_O_3_), and Caf (C_8_H_10_N_4_O_2_) were purchased from Tokyo Chemical Industry. Ethanol (EtOH), Methanol (MeOH), Hydrochloric acid (HCl), Acetic acid (C_2_H_4_O_2_, Glacial), and Chloroform (CHCl_3_) were received from RCI Labscan. Ammonium hydroxide (NH_4_OH) was obtained from J.T. Baker. Anhydrous disodium hydrogen phosphate (Na_2_HPO_4_), Sodiumchloride (NaCl), Potassiumdihydrogenorthophosphate (KH_2_PO_4_), and Potassiumchloride (KCl) were analytical grade and bought from KEMAUS. Polysorbate 20 (C_58_H_114_O_26_) and polysorbate 80 (C_64_H_124_O_26_) were purchased from Ajax Finechem. Phospholipon^®^ 90 G (Phosphatidylcholine) was manufactured by Lipoid GmbH, Ludwigshafen, Germany. Deionized water was obtained from Elix^®^ Millipore (Millipore, Molsheim, France), and MilliQ water was purified using a Milli-Q system (Milli-Q Q-Pod, Merck). Phosphate buffer solutions (PBS) were prepared from Na_2_HPO_4_, NaCl, KH_2_PO_4_, and KCl in Milli-Q water. Hair follicle dermal papilla cells (HFDPCs) were obtained from Cell Applications (San Diego, CA, USA). 3-(4,5-dimethylthiazol-2-yl)-2,5-diphenyltetrazolium bromide (MTT) and penicillin–streptomycin were purchased from Servicebio, Wuhan, Hubei, China. Dimethyl sulfoxide (DMSO) was purchased from Merck, Darmstadt, Germany. Fetal bovine serum (FBS) was purchased from Sigma-Aldrich, St. Louis, MO, USA. Dulbecco’s modified Eagle’s Medium (DMEM), trypsin-ethylenediaminetetraacetic acid, L-glutamine, non-essential amino acids, and phosphate-buffered saline (PBS) was purchased from Corning, NY, USA. 2,7-dichlorofluorescein diacetate (DCF-DA), dihydrotestosterone (DHT,) and minoxidil (MNX) were purchased from TCI (Tokyo, Japan). All compounds were used exactly as they were supplied without any extra purification.

### 3.2. Preparation of SiO_2_ Nanoparticle

The initial step involved dissolving 10 mL of NH_4_OH in a mixture of 95% ethanol and water (428 mL/60 mL) at 30 °C. Subsequently, 10 mL of TEOS was added and stirred at 900 rpm for two hours. The resulting precipitate was recovered by centrifugation (KUBOTA Model 6000, Osaka, Japan) at 15,000 rpm for 30 min and rinsed three times with distilled water and ethanol [40].

### 3.3. Preparation of SiO_2_@CTAB-SiO_2_ Core/Shell Nanoparticles

A solution of 0.15 g of CTAB was prepared by dissolving it in a mixture of 30 mL ethanol and 30 mL water. After stirring at 900 rpm for 30 min, 0.55 mL of ammonium hydroxide was added, followed by 0.1 g of SiO_2_ nanoparticles dispersed in 20 mL of distilled water. Then, 0.25 mL of TEOS was rapidly added, and the reaction persisted for six hours [40].

### 3.4. Synthesis of HMSNs via Selective Etching

The precipitate (SiO_2_@CTAB-SiO_2_ core/shell nanoparticles) was centrifuged at 15,000 rpm for 30 min, re-dispersed in 20 mL of deionized water, and stirred at 900 rpm for 10 h. The protocol for the etching process was derived from Chen et al. and involved the addition of CTAB 2.04 g and Na_2_CO_3_ 0.47 g to start a 10 h reaction at 50 °C [37]. The crude product was suspended in a methanol/HCl mixture of 50 mL methanol and 3 mL HCl, respectively, and refluxed at 80 °C for 24 h. The final product, HMSNs, was obtained through centrifugation at 15,000 rpm for 30 min and washed six times with distilled water [40]. Figure S2 shows the HMSN preparation.

### 3.5. Caf Loading into HMSNs (Caf@HMSNs)

A total of 0.1 g of Caf was dissolved in 50 mL of either water or ethanol, and 0.1 g of HMSNs was dispersed in the solution. The suspension underwent agitation for 24 h at 1000 rpm at room temperature. Subsequently, the Caf-loaded carrier was separated through filtration or centrifugation at 15,000 rpm for 10 min. Following this, it was dried in a vacuum at 60 °C for two hours after two rounds of cleaning with either 2 mL of ethanol or DI water [40]. The resulting product was named “Caf@HMSNs”.

### 3.6. Liposomal Dispersion Preparation (Lp-Caf@HMSNs and Derivatives)

#### 3.6.1. Preparing Stock Solutions

To prepare the Phospholipon^®^ 90 G stock solution, 0.773 g of Phospholipon^®^ 90 G was weighed into a glass vial and dissolved in 5 mL of a 2:1 *v*/*v* mixture of CHCl_3_ and MeOH. The solution was stirred until fully dissolved. For the cholesterol stock solution, 0.0618 g of cholesterol was weighed into a vial and dissolved in 8 mL of 2:1 *v*/*v* CHCl_3_ and MeOH was added, followed by stirring until completely dissolved. For polysorbate 20, 1.981 g of polysorbate 20 was weighed and dissolved in 2 mL of 2:1 *v*/*v* CHCl_3_ and MeOH with stirring. Similarly, with stirring, 2.112 g of polysorbate 80 was dissolved in 2 mL of 2:1 *v*/*v* CHCl_3_ and MeOH. Lastly, the oleic acid stock solution was prepared by weighing 0.991 g of oleic acid into a vial, adding 2 mL of a 2:1 *v*/*v* CHCl_3_ and MeOH, and stirring until dissolved.

#### 3.6.2. Synthesis of Ultradeformable Liposomes (ULp-Caf@HMSNs and Derivatives)

Hollow mesoporous silica nanoparticles loaded with Caf (0.01 g of HMSNs) were finely ground and divided into four test tubes, with each sample categorized by the type of polysorbate used in the formulation. The preparations were as follows:

- LpTW20-Caf@HMSNs: Caf-loaded HMSNs coated with a liposome containing polysorbate 20.
- LpTW80-Caf@HMSNs: Caf-loaded HMSNs coated with a liposome containing polysorbate 80.
- LpTW2080-Caf@HMSNs: Caf-loaded HMSNs coated with a liposome containing a 1:1 mixture of polysorbate 20 and polysorbate 80.
- Lp-Caf@HMSNs: Caf-loaded HMSNs coated with a liposome without polysorbate or oleic acid.
- Caf@HMSNs: Caf-loaded HMSNs without a liposome coating.
- Caf@LpTW20: Caf-loaded liposomes containing polysorbate 20.

The volumes of solutions used for each preparation are detailed in Table 5.

After adding each type of solution to the test tubes, gently rotate the test tubes while lightly blowing nitrogen gas to evaporate the solvent, forming a thin film around the inside of the test tubes. Once the solvent has completely evaporated, store the test tubes in a desiccator to remove moisture overnight. Then, add 3 mL of phosphate buffer solution (pH 7.4, temperature 80 ± 5 °C) to each test tube and mix with a mixer for 20 min; repeat this process twice. In the final step, add 4 mL of phosphate buffer solution and mix for 10 min. The final volume should be 10 mL. Ultrasonicate the mixture using a standard sonotrode (tip-diameter 3 mm, Hielscher UP200S, Teltow, Germany) at 40% amplitude for 20 min in an ice bath to disperse and reduce the size of the particles [25,29]. Collect the particles by centrifugation at 15,000 rpm for 30 min. The resulting product was stored in a cool, dry place. Figure S3 shows the preparation of Caf@HMSNs, Lp-Caf@HMSNs, and derivatives.

### 3.7. Characterization of Caf@HMSNs, ULp-Caf@HMSNs and Derivatives

The morphologies of all samples were examined using a transmission electron microscope (TEM; JEOL, JEM-2100 Plus, Tokyo, Japan) working at an accelerating voltage of 200 kV and a field-emission scanning electron microscope (FE-SEM; JEOL, JSM-7800F, Japan) with an accelerating voltage of 2–15 kV (Au-coated samples, coating thickness of 2 nm). Each sample was suspended in ethanol, sonicated, deposited onto a copper grid, and dried at 60 °C for 12 h for TEM examination. Fourier transform infrared spectroscopy was recorded using the KBr disc method (FT-IR; Nicolet iS50, Thermo Scientific, Waltham, MA, USA). Data were collected over 64 scans with a resolution of 4 cm^−1^, and the functional groups were examined in the 4000–400 cm^−1^ range. The average particle size, PDI, and zeta potential of all samples were measured using a dynamic light scattering (DLS) particle size analyzer (ZETASIZER Nano-ZS; Malvern Instruments, Worcestershire, UK) equipped with a 4 mW He–Ne laser at a 173° scattering angle. X-ray diffraction (XRD; Rigaku, MiniFlex600, Tokyo, Japan), operating at 40 kV and 30 mA, and scanning between 20° and 80°, was used to analyze the structures of the as-prepared samples. Cu-Kα radiation (k = 1.54 Å) was used for this purpose. A 3Flex instrument (Micromeritics, USA) was used to obtain the nitrogen sorption isotherms of the samples at –196 °C to ascertain their specific surface areas and pore size distributions. All samples underwent a 24 h degassing period at 250 °C (Smart VacPrep, Micromeritics, Norcross, GA, USA). The specific surface areas of the synthesized samples over the relative pressure (P/P_0_) range of 0.05–0.1 were determined using the Brunauer–Emmett–Teller (BET) method. The pore size distributions were established using the Barrett–Joyner–Halenda (BJH) method. The samples were evaluated for thermal stability and decomposition using a thermogravimetric analyzer (TGA; TGA 2, Mettler Toledo, Greifensee, Switzerland). In the 25–900 °C temperature range, the heating rate was 10 °C min^−1,^ and the nitrogen flow rate was 20 mL min^−1^.

### 3.8. High-Performance Liquid Chromatography (HPLC) Analysis

The Caf concentration in the supernatants was measured using HPLC (UFLC, Shimadzu, Kyoto, Japan) equipped with a C18 column (5 μm, 4.6 × 150 mm) using a UV detector at 280 nm, and the injection volume was 10 μL. The freshly prepared 50:50 *v*/*v* acetic acid and water made up the isocratic mobile phase, which was pumped at a rate of 0.8 mL min^−1^. The mixture was filtered through a 0.2 μm filter (VertiClean™ NYLON) and degassed using sonication for 30 min. All separation was carried out at ambient temperature.

### 3.9. Entrapment Efficiency (%EE) and Loading Capacity (%LC)

The entrapment efficiency (%EE) and loading capacity (%LC) of the Caf@HMSNs, ULp-Caf@HMSNs, and derivatives were determined using the following method. In brief, the solid particles were centrifuged at 15,000 rpm for 30 min to separate the supernatant. After one water wash, the solid particles were dried in an oven set to 60 °C overnight and stored in a desiccator. The supernatants were filtered through a Millipore 0.2 μm filter before each measurement. HPLC analysis was used to determine the concentration. Equations (1) and (2) were used to obtain the %EE and %LC [29,105].

%EE = (C_f_/C_i_) × 100. (1)

Cf denotes the Caf encapsulated in HMSNs, while Ci denotes the original amount added to the HMSNs.

%LC = (C_f_/H) × 100. (2)

The amount of Caf@HMSNs is denoted by H, while the amount of Caf entrapped in the HMSNs is represented by C_f_.

### 3.10. Drug Release Behaviour

For evaluating drug-release behavior, the synthesized samples (net weight of HMSNs 3.0 mg) were re-dispersed in glass bottles holding 30 mL of PBS (pH = 7.4) to examine the drug release profile of Caf@HMSNs, ULp-Caf@HMSN, and derivatives (performed under sink conditions). The next step was agitation at 100 rpm at 37.0 ± 0.5 °C. Then, 100 μL aliquots of the medium were taken at 0, 20, 40, 60, 80, 100, 120, 240, 300, 360, 420, and 480 min. Before every measurement, the sample was diluted appropriately with water. The supernatants were filtered via a Millipore 0.2 μm filter before each measurement. HPLC analysis was used to determine the concentration.

The mass of Caf (Mc) released in time t was calculated using the concentration of Caf in the release medium. The cumulative amount (Mc′) was adjusted to account for the Caf content of the previously removed samples [73]. Equation (3) was used to determine the percent release:

% Release = (Mc′/Cf) × 100.(3)

The amount of Caf entrapped in the HMSNs is represented by Cf.

Equation (4) presents the zero-order release model used to calculate the drug release profile [74]:

Mc′ = k_0_t. (4)

Equation (5) presents the first-order release model used to calculate the drug release profile [106,107]:

ln (Cf − Mc′) = ln Cf − k_1_t. (5)

Equation (6) presents the Higuchi release model used to calculate the drug release profile [76]:

Mc′ = k_H_t^0.5^. (6)

Equation (7) presents the Korsmeyer-Peppas release model used to calculate the drug release profile [75]:

(Mc′/Cf) = kt^n^. (7)

Cf stands for the total amount of Caf entrapped in the HMSNs, Mc′ is the cumulative amount of Caf released at time t, t represents the elapsed time, and k (k_0_, k_1_, k_H_, or k) is the release rate constant [107]. The models above describe the release of Caf, a water-soluble substance, from HMSNs, which typically release their cargo at a rate proportional to the amount inside the porous structure [73].

### 3.11. Skin Penetration Study

The in vitro permeation of LpTW20-Caf@HMSNs, LpTW80-Caf@HMSNs, LpTW2080-Caf@HMSNs, Lp-Caf@HMSNs, Caf@HMSNs, and Caf@LpTW20 through porcine skin was evaluated using a Franz-type diffusion cell (PermeGear, Hellertown, PA, USA), following a previously published method with some modifications [77]. Fresh porcine skin was harvested from a pig abattoir in Pathum Thani province, with the SC carefully separated from the underlying tissue and stored in 0.9% NaCl solution at 4 °C until use. The SC was cut into 2.0 cm × 2.0 cm sections and positioned between the donor and receptor chambers of the diffusion cell, with the receptor chamber containing 15 mL PBS (pH 7.4). The receptor medium was stirred continuously and maintained at 37.0 ± 0.5 °C using a water jacket. The synthesized samples (net weight of HMSNs 1.5 g) were dispersed in 1 mL PBS (pH 7.4) and placed in the donor chamber. For 6 h, 0.5 mL of release medium was removed at designated intervals and replaced with fresh PBS. The medium was filtered through a 0.2 μm Millipore filter (VertiClean™ NYLON), and HPLC analyzed the Caf content at 280 nm. The remaining Caf in the skin after 24 h was also evaluated. After removing the porcine skin, it was cleaned with distilled water, cut into 1.5 cm circles, and soaked in 1 mL of water overnight. The Caf was extracted via ultrasonication for two hours, and the solution was centrifuged at 15,000 rpm for 15 min. HPLC analyzed the supernatant to determine the residual Caf [108]. Permeation results are expressed as the cumulative amount per area (μg cm^−2^) versus time (hours), as the steady-state flux (J_ss_) (μg cm^−2^ h^−1^), and as the apparent permeability coefficient (Papp) (×10^−1^ cm s^−1^).

### 3.12. Confocal Laser Scanning Microscopy (CLSM) Study

Fluorescein isothiocyanate (FITC) was used as a model for Caf to observe the interaction between particles and the skin surface. Porcine skin samples were treated with FITC@HMSNs and LpTW20-FITC@HMSNs, where the particles’ outer layer was stained with Rhodamine B. The in vitro skin permeation was evaluated using the previously described Franz-type diffusion cell method. Skin samples were collected at 1 and 6 h, rinsed with water, and gently dried with a Kimwipe before freezing at −20 °C for fixation. The samples were then sliced into 10 µm thick sections using a cryostat (Model CM 1950, Wetzlar, Germany) maintained at −20 °C, while the specimen head was held at −25 °C. A confocal laser scanning microscope (CLSM) (LSM 800, Zeiss, Jena, Germany) was used to visualize the porcine skin cross-sections and to measure the depth of particles that penetrate through the layer of porcine skin. The system employed argon and diode lasers to independently track FITC and Rhodamine B within the skin tissue. FITC was excited at 488 nm, emitting a green signal, while Rhodamine B was excited at 568 nm, emitting a red signal. The staining of the particles’ outer layer aided in locating them within the SC and tracking FITC release. Images were captured using a 10× EC-Plan Neofluar objective lens, with eight scans averaged for each image [109,110].

### 3.13. MTT Assay for Cell Viability Analysis

Hair follicle dermal papilla cells (HFDPCs) were obtained from Cell Applications (CA, USA). HFDPCs were cultured with DMEM supplement mixed with (10%, *v*/*v*) FBS, (1%, *v*/*v*) L-glutamine, (1%, *v*/*v*) non-essential amino acid, and (1%, *v*/*v*) penicillin-streptomycin under 5%CO_2_/95% humidified at 37 °C. The MTT test was used to measure mitochondrial activity and cell viability to evaluate the effect of pure Caf, HMSNs, Caf@HMSNs, and LpTW20-Caf@HMSNs on cytotoxicity. In 96-well plates (BD BioCoats, Japan), HFDPCs were seeded at a density of 1 × 10^4^ cells per well and cultured for a full day. After that, for 24 h, HFDPCs were treated with pure Caf, HMSNs, Caf@HMSMs, and LpTW20-Caf@HMSNs with the actual amount of Caf 0.0125, 0.025, 0.05, 0.10, 0.15, 0.20, and 0.25 mg mL^−1^. After mixing with 5 mg mL^−1^ MTT dissolved in PBS (10 μL/well), the cells were incubated for an additional three hours. A microtiter plate reader (FLUOstar Omega, Germany) was used to measure the absorbance at 570 nm after the resultant MTT formazan was dissolved in 100 μL of DMSO. Equation (8) was used to calculate the % Cytotoxicity or cell proliferation [16].

(8) $$\%\;\mathrm{Cytotoxicity}\;\mathrm{or}\;\mathrm{cell}\;\mathrm{proliferation}=\frac{\mathrm{Absorbance}\;\mathrm{value}\;\mathrm{of}\;\mathrm{treatment}\times 100}{\mathrm{The}\;\mathrm{absorbance}\;\mathrm{value}\;\mathrm{of}\;\mathrm{the}\;\mathrm{control}}.$$

### 3.14. Study of Cell Morphology and Aggregation Behavior

The methodology used to determine the behavior of cell aggregation was that of Kiratipaiboon et al. [111]. Cells were seeded at a density of 8 × 10^3^ cells per well in 24-well plates and incubated overnight for cell attachment. The cells were treated with 0.0125 and 0.025 mg mL^−1^ of Caf (pure Caf, HMSNs, Caf@HMSMs, and LpTW20-Caf@HMSNs) for 72 h. The cell morphology was monitored and documented during the treatment period at 0, 24, 48, and 72 h. Additionally, the aggregation behavior of the cells was evaluated at the 72 h mark. The cellular morphology and aggregation behavior were captured using a phase-contrast microscope (ECLIPSE Ts2, Nikon, Tokyo, Japan) for further analysis.

### 3.15. DCF-DA Reactive Oxygen Species (ROS) Assay

HFDPCs were seeded at a density of 2.5 × 10^4^ cells per well in 24-well plates and incubated for 24 h. The cells were then treated with 1 μM DHT, along with equal concentrations of various samples (0.0125 and 0.025 mg mL^−1^ of pure caffeine, HMSNs, Caf@HMSNs, and LpTW20-Caf@HMSNs), as well as 1 μM MNX, for 24 h at 37 °C in a CO_2_ incubator. After treatment, the cells were washed twice with DPBS and stained with 10 μM 2,7-Dichlorofluorescein diacetate (DCF-DA) for 15 min. Following staining, the cells were washed again with DPBS, and 200 μL of DPBS was added to each well for fluorescence measurement [95].

### 3.16. Statistical Analysis

All data were statistically analyzed and presented as the mean ± standard deviation (SD) from three independent experiments. A one-way analysis of variance (ANOVA) was used, followed by Tukey’s Multiple Comparison Test to assess differences between groups. Results with *p*-values less than 0.05 or 0.01 were considered statistically significant.

## 4. Conclusions

In conclusion, synthesizing and characterizing HMSNs, ULp-HMSNs, and their derivatives demonstrated their potential as effective drug delivery systems for Caf. FT-IR analysis confirmed the successful coating of HMSNs with liposomes, while SEM and TEM images showed particles ranging from 140 to 183 nm, which are ideal for enhanced skin permeability. Zeta potential measurements indicated good colloidal stability, and N_2_ sorption confirmed the mesoporous structure, with an average pore size of 3.6 nm and a pore volume of 1.11 mL g^−1^. The specific surface area, measured by BET, was 1044 m^2^ g^−1^ and the particles, less than 200 nm in size, exhibited a larger pore volume than typical MSNs. The diffraction pattern also displayed a characteristic silica peak at 2θ = 23°, indicating the hollow core–shell structure. Regarding drug loading capacity, HMSNs facilitated higher Caf loading, achieving 26.47% LC and 36.01% EE, which was 5–6% better than Caf@LpTW20. The release profiles showed that Caf@HMSNs and Caf@LpTW20 had rapid drug release rates, while LpTW20-Caf@HMSNs, LpTW80-Caf@HMSNs, LpTW2080-Caf@HMSNs, and Lp-Caf@HMSNs exhibited more controlled release, with the liposome coating slowing down Caf release by sealing the pores in the HMSNs. This combination ensures gradual drug release, providing sustained delivery and reducing the need for frequent doses. Among the various formulations, LpTW20-Caf@HMSNs exhibited the highest caffeine permeation. This is attributed to the high hydrophilicity of polysorbate 20, a component of the formulation, and its small molecular structure, which enhances lipid barrier disruption. This formulation also demonstrated controlled release properties, with 65% of Caf released over eight hours. CLSM imaging confirmed that liposome-coated particles penetrate deeper into the skin (310 µm) than non-liposomal particles (270 µm), enhancing drug permeation across skin layers. Additionally, CLSM confirmed the release of the encapsulated compound from HMSNs into the hair follicle, revealing a higher fluorescence intensity at 488 nm and 568 nm within the follicle compared to the surrounding skin. This finding indicates that incorporating FITC into the particles improves targeted delivery to the specific cells while limiting release in non-target regions. The non-cytotoxic nature of HMSNs, confirmed by cell viability studies, supports their safety for clinical use. LpTW20-Caf@HMSNs showed even greater cell viability than pure Caf and Caf@HMSNs, indicating that the liposome coating enhances biocompatibility without increasing cellular toxicity. In addition, Caf@HMSNs and LpTW20-Caf@HMSNs significantly reduced ROS levels in DHT-damaged HFDPCs, suggesting a promising alternative to MNX for promoting hair follicle growth and reducing hair loss without increased oxidative stress. Overall, LpTW20-Caf@HMSNs represent a promising approach for targeted, controlled drug delivery in alopecia treatment.

## 5. Patents

Portions of this content have been submitted for a petty patent application in Thailand under application number 2403002357.

## Figures and Tables

**Table 1:** The zeta potential, polydispersity index (PDI), and average particle size (from the DLS technique) of different HMSNs, ULp-HMSNs, and derivatives.

Name	Size (nm)	PDI	Zeta (mV)
LpTW20-HMSNs	182.1 ± 15.96	0.519 ± 0.08	−33.1 ± 4.51
LpTW80-HMSNs	170.4 ± 24.98	0.370 ± 0.07	−33.7 ± 0.61
LpTW2080-HMSNs	170.9 ± 6.29	0.425 ± 0.19	−32.6 ± 8.62
Lp-HMSNs	175.8 ± 17.61	0.421 ± 0.09	−30.1 ± 2.48
HMSNs	152.4 ± 6.91	0.514 ± 0.18	−26.2 ± 2.43
LpTW20	78.96 ± 17.34	0.455 ± 0.07	−33.4 ± 0.53

Each value represents the mean ± SD (*n* = 3).

**Table 2:** The percentages of EE and LC of Caf loading in HMSNs with water and ethanol as solvent.

Name	Solvent	%EE	%LC	Amount of Caf in 0.1 g Particle (mg)
Caf@HMSNs	Water	36.01± 0.13	26.47 ± 0.06	36.01 ± 0.13
Caf@HMSNs	EtOH	32.10 ± 0.24	24.32 ± 0.11	32.10 ± 0.24
Caf@LpTW20	Water	30.87 ± 0.17	−	30.87 ± 0.17

Each value represents the mean ± SD (*n* = 3).

**Table 3:** The release kinetic data for the Caf released from Caf@HMSNs, ULp-Caf@HMSNs, and derivatives.

Formulation	% Release at Eight Hours	Ct (mg)	Zero-Order Model		First-Order Model		Higuchi Model		Korsmeyer-Peppas Model		
			k	R^2^	k	R^2^	k	R^2^	k	R^2^	n
LpTW20-Caf@HMSNs	65 ± 4.32	23.4 ± 1.55	0.11	0.8713	0.48	0.9853	2.91	0.9921	0.11	0.9336	0.3453
LpTW80-Caf@HMSNs	71 ± 3.51	25.5 ± 1.26	0.12	0.8749	0.52	0.9819	3.42	0.9916	0.11	0.9128	0.3596
LpTW2080-Caf@HMSNs	56 ± 2.79	19.8 ± 1.01	0.08	0.8358	0.34	0.9870	2.11	0.9886	0.22	0.9397	0.2491
Lp-Caf@HMSNs	42 ± 6.47	14.4 ± 2.33	0.07	0.8469	0.26	0.8547	1.84	0.9813	0.11	0.9514	0.3555
Caf@HMSNs	94 ± 1.76	33.9 ± 0.63	0.14	0.7661	0.37	0.9593	3.74	0.9744	0.19	0.9347	0.2363
Caf@LpTW20	83 ± 1.12	25.5 ± 0.34	0.09	0.5692	0.47	0.9878	2.73	0.9881	0.26	0.9366	0.2773

Each value represents the mean ± SD (*n* = 3).

**Table 4:** In Vitro permeation profiles of LpTW20-Caf@HMSNs, LpTW80-Caf@HMSNs, LpTW2080-Caf@HMSNs, Lp-Caf@HMSNs, Caf@HMSNs, and Caf@LpTW20 in PBS at pH 7.4.

Formulation	Cumulative Amount per Area at Six Hours (μg cm^−2^)	Cumulative Amount in Porcine Skin (μg)	Steady-State Flux (J_ss_) (μg cm^−2^ h^−1^)	Permeability Coefficient (P_app_) (×10^−5^ cm s^−1^)
LpTW20-Caf@HMSNs	368.06 ± 13.35	60.25 ± 6.03	61.88 ± 4.23	11.45 ± 7.83
LpTW80-Caf@HMSNs	340.65 ± 20.55	61.40 ± 5.15	54.89 ± 3.73	10.16 ± 6.92
LpTW2080-Caf@HMSNs	359.33 ± 28.41	60.91 ± 7.23	59.12 ± 3.49	10.95 ± 6.46
Lp-Caf@HMSNs	221.02 ± 32.90	66.78 ± 6.61	32.87 ± 4.37	6.09 ± 7.91
Caf@HMSNs	168.79 ± 20.10	59.07 ± 5.66	24.18 ± 3.93	4.48 ± 1.47
Caf@LpTW20	39.56 ± 3.14	25.79 ± 4.96	6.11 ± 0.58	1.13 ± 0.11

Each value represents the mean ± SD (*n* = 3).

**Table 5:** The amount of ingredients used to prepare Lp-Caf@HMSNs, ULp-Caf@HMSNs, and its derivatives.

Name	Volume of Phospholipon^®^ 90 G (μL)	Volume of Cholesterol (μL)	Volume of Polysorbate 20 (μL)	Volume of Polysorbate 80 (μL)	Volume of Oleic Acid (μL)
LpTW20-Caf@HMSNs	250	500	100	-	50
LpTW80-Caf@HMSNs	250	500	-	100	50
LpTW2080-Caf@HMSNs	250	500	50	50	50
Lp-Caf@HMSNs	250	500	-	-	-

## Data Availability

Data are contained within the article or Supplementary Materials.

## Supplementary Materials

The following supporting information can be downloaded at: https://www.mdpi.com/article/10.3390/ijms252212170/s1. References [112,113] are cited in Supplementary Materials.

## Abbreviations

AGA	Androgenetic alopecia
DHT	Dihydrotestosterone
HFDPCs	Hair follicle dermal papilla cells
HMSNs	Hollow mesoporous silica nanoparticles
MSNs	Mesoporous silica nanoparticles
Caf	Caffeine
Caf@HMSNs	Caffeine-loaded hollow mesoporous silica nanoparticles
Lp-Caf@HMSNs	Caffeine-loaded hollow mesoporous silica nanoparticles coated with liposomes
ULs	Ultradeformable liposomes
ULp-Caf@HMSNs	Caffeine-loaded hollow mesoporous silica nanoparticles coated with ultradeformable liposomes
LpTW20-Caf@HMSNs	Caffeine-loaded HMSNs coated with a liposome containing polysorbate 20. (coated with ULs)
LpTW80-Caf@HMSNs	Caffeine-loaded HMSNs coated with a liposome containing polysorbate 80. (coated with ULs)
LpTW2080-Caf@HMSNs	Caffeine-loaded HMSNs coated with a liposome containing a 1:1 mixture of polysorbate 20 and polysorbate 80. (coated with ULs)
Caf@HMSNs	Caffeine-loaded HMSNs without a liposome coating.
Caf@LpTW20	Caffeine-loaded liposomes containing polysorbate 20. (coated with ULs)
ULpTW-HMSNs	HMSNs coated with a liposome containing polysorbate. (coated with ULs)
LpTW20-HMSNs	HMSNs coated with a liposome containing polysorbate 20. (coated with ULs)
LpTW80-HMSNs	HMSNs coated with a liposome containing polysorbate 80. (coated with ULs)
LpTW2080-HMSNs	HMSNs coated with a liposome containing a 1:1 mixture of polysorbate 20 and polysorbate 80. (coated with ULs)
Lp-HMSNs	HMSNs coated with a liposome without polysorbate or oleic acid.
FITC	Fluorescein isothiocyanate
FITC@HMSNs	FITC-loaded hollow mesoporous silica nanoparticles
LpTW20-FITC@HMSNs	FITC-loaded HMSNs coated with a liposome containing polysorbate 20. (coated with ULs)
CTAB	Cetyltrimethylammonium bromide
DCF-DA	2,7-dichlorofluorescein diacetate
DLS	Dynamic light scattering
DMEM	Dulbecco’s modified Eagle’s Medium
DMSO	Dimethyl sulfoxide
FBS	Fetal bovine serum
J_ss_	Steady-state flux
MNX	Minoxidil
P_app_	Apparent permeability coefficient
PBS	Phosphate-buffered saline
ROS	Reactive Oxygen Species
TEOS	Tetraethoxysilane
TMOS	Tetramethoxysilane
EtOH	Ethanol
MeOH	Methanol
TEM	Transmission electron microscopy
SEM	Scanning electron microscopy
CLSM	Confocal laser scanning microscopy
HPLC	High-performance liquid chromatography
FT-IR	Fourier transform infrared spectroscopy
XRD	X-ray diffraction
%EE	% Entrapment efficiency
%LC	% Loading capacity
MTT	(3-(4,5-dimethylthiazol-2-yl)-2,5-diphenyltetrazolium bromide)
PDI	Polydispersity index
IUPAC	The International Union of Pure and Applied Chemistry
BET	Brunauer–Emmett–Teller
BJH	Barrett–Joyner–Halenda
TGA	Thermogravimetric analysis
Td_10_	10% weight-loss temperatures
SD	Standard deviation
HLB	Hydrophilic–lipophilic balance
SC	Stratum corneum
