# Supplementary material for: Nanotechnology-Driven Delivery of Caffeine Using Ultradeformable Liposomes-Coated Hollow Mesoporous Silica Nanoparticles for Enhanced Follicular Delivery and Treatment of Androgenetic Alopecia
Source: Int J Mol Sci. 2024 Nov 13;25(22):12170. doi: 10.3390/ijms252212170 (PMC11595114; doi:10.3390/ijms252212170)
Supplement: Supplementary file 1 [file ijms-25-12170-s001.zip › ijms-3303304-supplementary.pdf]

Nanotechnology-Driven Delivery of Caffeine Using Ultradeformable Liposomes-Coated Hollow Mesoporous Silica Nanoparticles for Enhanced Follicular Delivery and Treatment of Androgenetic Alopecia

Nattanida Thepphankulngarm [1], Suwisit Manmuan [2], Namon Hirun [1] and Pakorn Kraisit [1,*]

[1] Thammasat University Research Unit in Smart Materials and Innovative Technology for Pharmaceutical Applications (SMIT-Pharm), Faculty of Pharmacy, Thammasat University, Pathumthani 12120, Thailand; nattanida.t@gmail.com (N.T.); namon.hi@tu.ac.th (N.H.)
[2] Division of Pharmacology and Biopharmaceutical Sciences, Faculty of Pharmaceutical Sciences, Burapha University, Chonburi 20131, Thailand; suwisit@go.buu.ac.th

* Correspondence: pakorn54@tu.ac.th

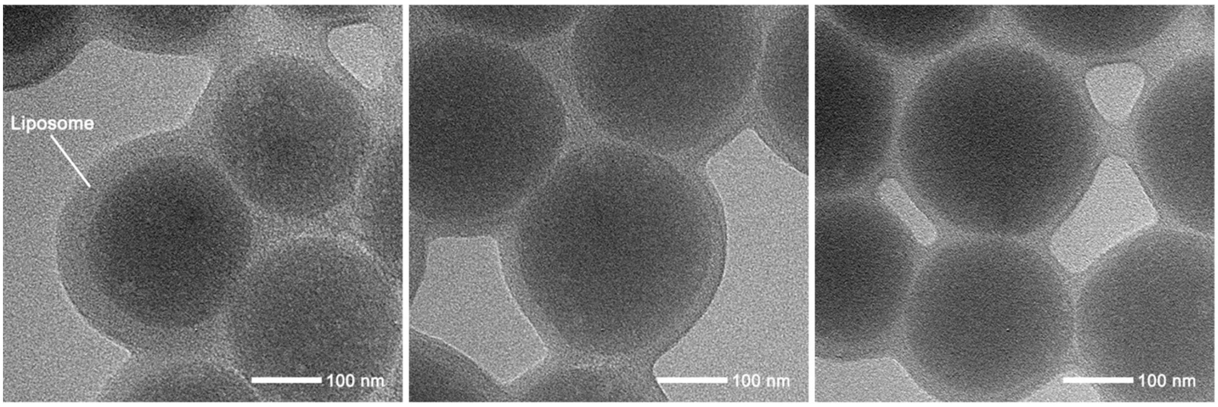

Figure S1. TEM images of LpTW20-Caf@HMSNs

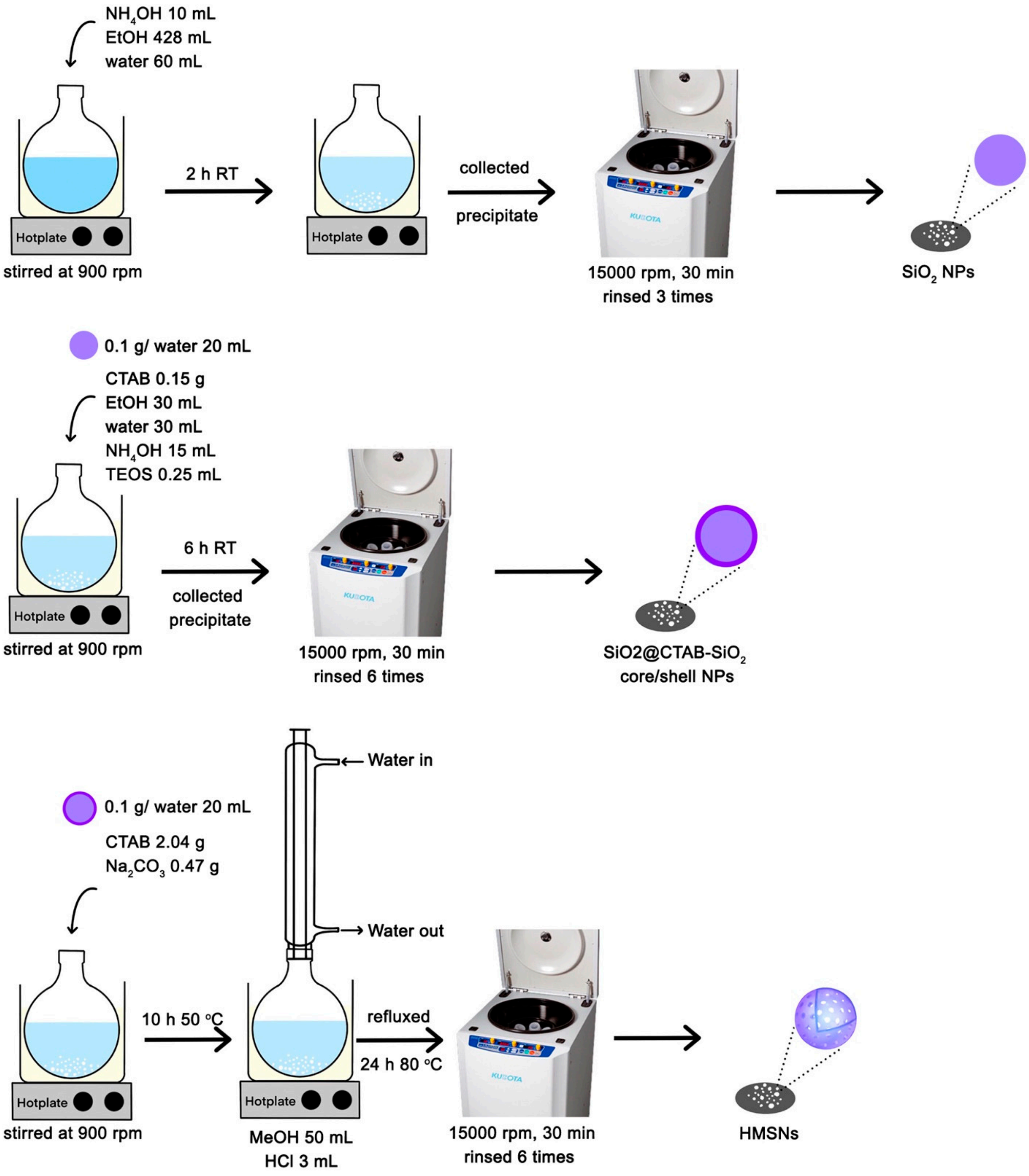

Figure S2. The preparation of HMSNs.

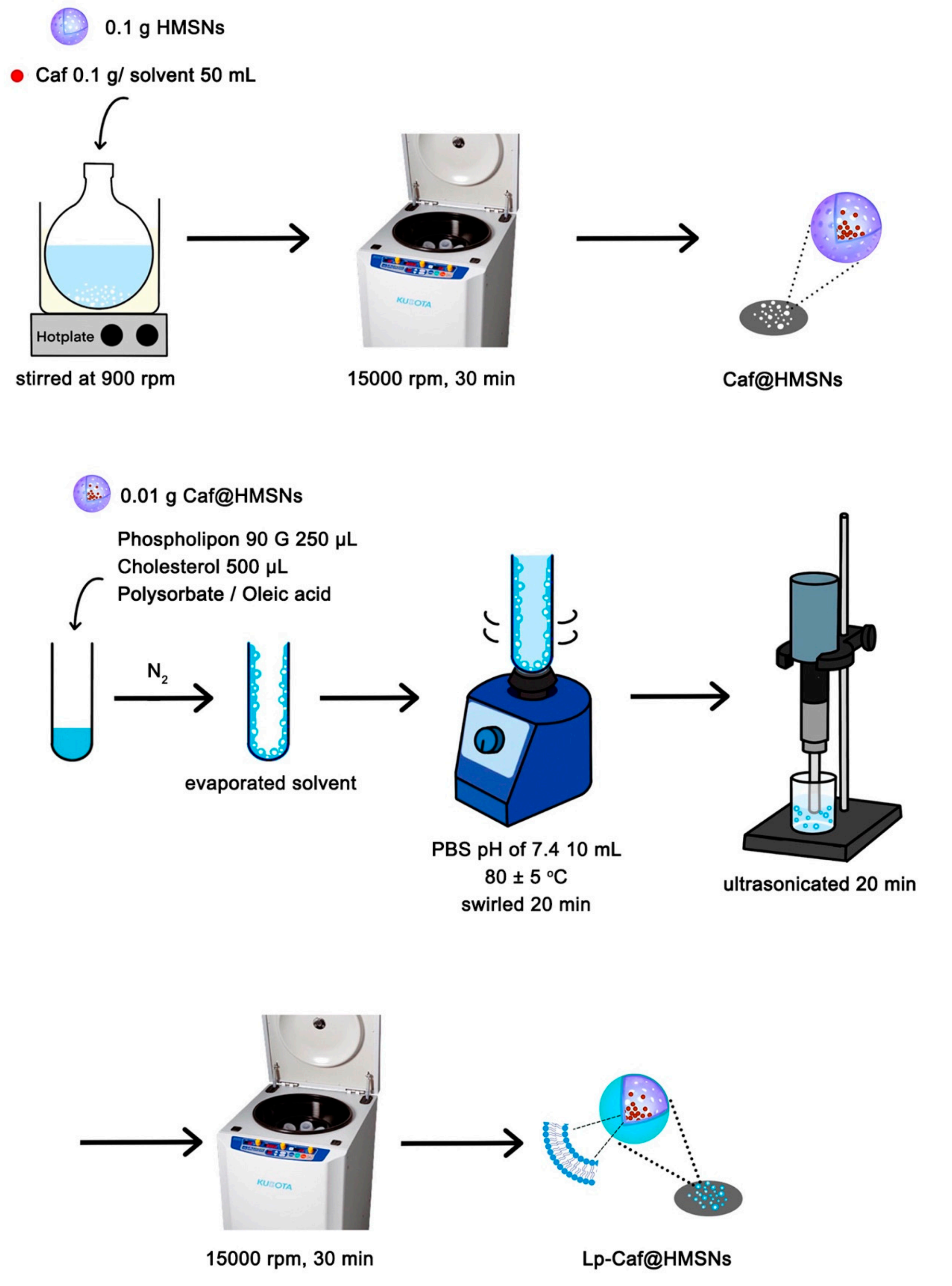

Figure S3. The preparation of Caf@HMSNs, ULp-Caf@HMSNs, Lp-Caf@HMSNs and derivatives.

Table S1 The ability to load Caf into nanoparticles (NPs) in this study compared to other works.

Type of the nanoparticles	%LC	Reference
CAF@silica NPs	20%	112
CAF@SBA-15	23%	112
Poly-ε-caprolactone (PCL) NPs	7.6-10.4%	113
Poly-ε-caprolactone (PCL) NPs	25%	113
**HMSNs**	**26.47%**	**This work**

112. N. Liédana, E. Marín, C. Téllez, J. Coronas, One-step encapsulation of caffeine in SBA-15 type and non-ordered silicas, Chemical Engineering Journal 223 (2013) 714-721.

113. D. Massella, A. Ancona, N. Garino, V. Cauda, J. Guan, F. Salaun, A.A. Barresi, A. Ferri, Preparation of bio-functional textiles by surface functionalization of cellulose fabrics with caffeine loaded nanoparticles, IOP Conference Series: Materials Science and Engineering 460(1) (2018) 012044.
